# Tailored Metal‐Organic Framework‐Based Enzyme Hybrids: Immobilization Strategies, Improved Performance, and Biological Applications

**DOI:** 10.1002/smsc.202500222

**Published:** 2025-07-10

**Authors:** Xiang Xu, Jiacheng Wan, Jun Sun, Lina Wu, Jianping Lei

**Affiliations:** ^1^ State Key Laboratory of Microbial Technology School of Food Science and Pharmaceutical Engineering Nanjing Normal University Nanjing 210023 China; ^2^ State Key Laboratory of Analytical Chemistry for Life Science School of Chemistry and Chemical Engineering Nanjing University Nanjing 210023 China

**Keywords:** cancer therapy, enzyme, immobilization, metal‐organic frameworks, protein delivery

## Abstract

Natural enzymes can efficiently and selectively catalyze various chemical reactions, but are limited by inherent fragility. Assembled via tailored metal nodes and organic ligands, metal‐organic frameworks (MOFs) offer unique advantages for enzyme immobilization due to their customizable structures. According to the enzymes spatial position within MOF's structure, MOF‐based enzyme immobilization strategies can be generally categorized into surface attachment, pore infiltration, and encapsulation. When enzymes are positioned close to MOF's surface, MOFs can offer limited protection. While deeper embedding provides stronger protection, it hinders the diffusion of substrates, products, and cofactors, thereby limiting catalytic efficiency. With advanced understanding of MOF synthesis, precise design and modulation of MOF structures enable improved performance of MOF‐enzyme hybrids. According to MOF's diversity, precise design strategies of MOFs can be classified as surface microenvironment modulation, pore size and volume design, morphology tuning, and defect engineering. These strategies significantly optimize the enzymatic microenvironment, enzyme loading efficacy, and mass transfer, thereby improving the performance of MOF‐enzyme hybrids. This review summarizes MOF‐based enzyme immobilization strategies, explores precise designs overcoming various limitations, and highlights their applications in biosensing, biocatalysis, stimulus‐responsive delivery, and cancer therapy. Additionally, the potentials of MOFs with enhanced enzyme stability and functionality in broader applications are explored.

## Graphical abstract

Metal‐organic frameworks (MOFs) offer versatile platforms for enzyme immobilization to overcome their inherent fragility. However, challenges such as restricted conformation and mass transfer limitations remain. Advances in precise MOF design can help overcome these obstacles, expanding their applicability across diverse fields, highlighting the potential of MOF‐enzyme hybrids as efficient hosts for encapsulating biological macromolecules.

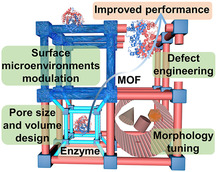

## 1 Introduction

With diverse and distinctive 3D structures evolved over millions of years, enzymes can catalyze a wide range of chemical reactions,^[^ ^1^, ^2^ ^]^ playing essential roles in almost all life processes such as growth, metabolism, and replication.^[^ ^3^ ^]^ Benefiting from their high efficiency, specificity, selectivity, and diversity in reaction catalysis,^[^ ^4^ ^]^ enzymes have been utilized in many fields, including biosynthesis, biotransformation, biosensing, and biofuel,^[^ ^5^ ^]^ where synthetic catalysts may be limited by low reaction rate and poor product selectivity.^[^ ^6^ ^]^ Additionally, a combination of multiple enzymes for cascade catalysis allows one‐pot synthesis of high‐value‐added products, achieving green and low‐cost chemistry.^[^ ^7^ ^]^ Though promising, the extended application of enzymes is hindered by their susceptibility to environmental factors such as pH, temperature, organic solvents, surfactants, and pressure.^[^ ^8^ ^]^

To improve their performance, a variety of porous materials have been researched for the immobilization of enzymes, such as hydrogels, mesoporous silica, metal‐organic frameworks (MOFs), and so on.^[^ ^9^, ^10^, ^11^ ^]^ MOFs are a class of organic–inorganic hybrid materials linked by coordination interactions between organic ligands and metal nodes.^[^ ^12^ ^]^ Due to their modular construction and tunable nature (metal nodes and ligands), MOFs can be designed with desired surface groups, pore environments, and morphology.^[^ ^13^ ^]^ Given these tailored features, MOFs are viewed as suitable platforms for engineering the surrounding microenvironments of enzymes, protecting enzymes from severe conditions.^[^ ^14^ ^]^ Currently, strategies to construct MOF‐enzyme hybrids can be generally divided into surface attachment, pore infiltration, and encapsulation.^[^ ^15^ ^]^ For surface attachment, it refers to attaching enzymes to the surface of MOFs through covalent bonds or other interactions. This loading method does not affect the structures of either the enzyme or the MOF, but the protection the MOF provides to the enzyme is limited. Pore infiltration means immersing enzymes into the presynthesized pores of MOFs. This method is relatively simple to perform and can provide effective protection for the enzymes. However, for enzymes of different sizes, it is necessary to design and synthesize mesoporous MOFs with varying pore sizes accordingly.^[^ ^16^ ^]^ Encapsulation refers to incorporating enzymes into the MOF during its synthesis. Encapsulation is simple and effective without requiring extensive design of the MOF. However, the MOF may restrict the passage of substrates, products, and cofactors, thereby limiting the catalytic efficiency of the enzyme.^[^ ^17^ ^]^ Overall, different enzyme immobilization methods have their own advantages and disadvantages, and the selection should be flexible based on specific applications.^[^ ^18^ ^]^

**Figure 1:**
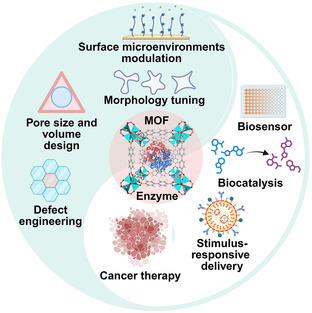
Schematic illustration of precisely designed MOF‐enzyme hybrids with enhanced performance for biological applications.

Despite the promising potential of MOFs‐immobilized enzymes, challenges such as restricted conformation of enzymes and limited mass transfer remain.^[^ ^19^ ^]^ Luckily, with the advancement of materials science and a deeper understanding of MOFs synthesis, the precise design and modulation of MOFs have become feasible.^[^ ^20^ ^]^ To further enhance the catalytic performance of immobilized enzymes, MOFs design strategies include tuning the surface polarity, expanding pore size, enlarging cavity volume, and adjusting MOF morphology that have been developed.^[^ ^21^, ^22^ ^]^ Overall, these strategies primarily aim to optimize the enzymatic microenvironment, increase enzyme loading capacity, and improve mass transport. The tunable composition of MOFs, along with their facile post‐synthetic modification, makes them highly compatible with these design principles.^[^ ^23^ ^]^ By meticulously selecting the metal ions and organic ligands for MOFs construction, precisely optimizing synthesis conditions, and employing post‐synthetic modifications, MOFs with well‐defined morphology, size, and porosity can be obtained.^[^ ^24^, ^25^ ^]^ This tailored design approach allows for the synthesis of MOFs as enzyme carriers specifically adapted to the intrinsic properties of target enzymes, thereby maximizing the catalytic efficiency.^[^ ^26^ ^]^ In this review, we summarize MOF‐based enzyme immobilization strategies and discuss their corresponding advantages and limitations. Precise designs of MOFs, such as surface microenvironments modulation, pore size and volume design, morphology tuning, and defect engineering, are also outlined to offer inspiration for future research (**Figure** **1**). These designs show great potential in applications like biosensing, biocatalysis, stimulus‐responsive delivery, and cancer therapy. Based on these understandings, possible future directions in the development and advanced applications of MOF‐enzyme hybrid are proposed.

## 2 Location‐Specific Enzyme Immobilization Strategies

**Figure 2:**
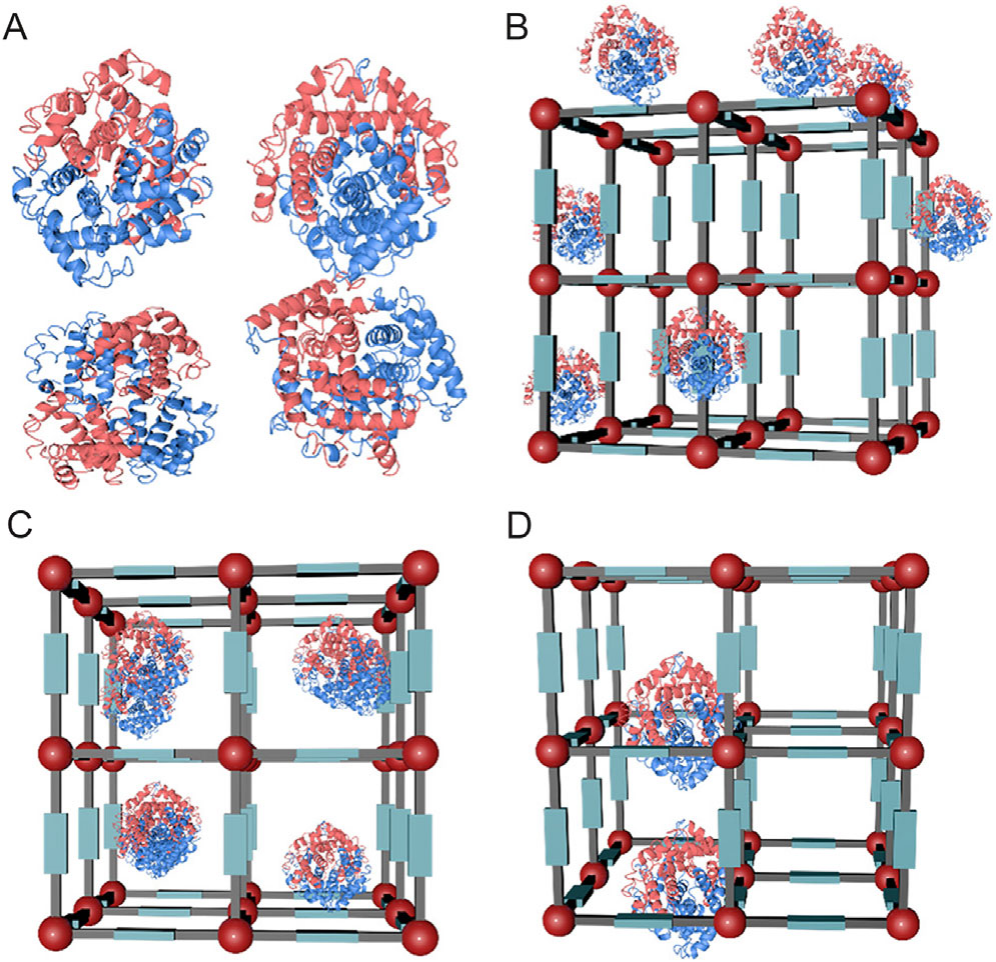
Schematic illustration of immobilization types of MOF‐enzyme hybrids: A) free enzyme, B) surface attachment, C) pore infiltration, and D) encapsulation.

The primary goal of using MOFs for enzyme immobilization is to preserve the catalytic activity of enzymes while enhancing their recyclability.^[^ ^27^ ^]^ The structure of enzymes is determined by amino acid sequences, hydrogen bonds, salt bridges, and hydrophobic forces. By avoiding direct contact between the enzymes and the unfavorable environment, MOF can effectively protect these chemical bonds from being destroyed, allowing the enzymes to maintain their stable tertiary structure. Among the commonly used immobilization strategies, i.e., surface attachment, pore infiltration, and encapsulation,^[^ ^28^, ^29^, ^30^ ^]^ deeper incorporation of enzymes within the MOF's structure generally offers greater protection (**Figure** **2**). However, this increased protection can hinder substrate accessibility, leading to reduced catalytic efficiency. Additionally, while higher enzyme loading can boost overall product yield, it may also dilute the local substrate concentration relative to single enzyme molecules, further decreasing the average catalytic efficiency.^[^ ^31^ ^]^ The following sections will highlight the advantages of various immobilization methods and discuss the improvements designed to overcome their respective limitations.

### 2.1 Surface Attachment

**Figure 3:**
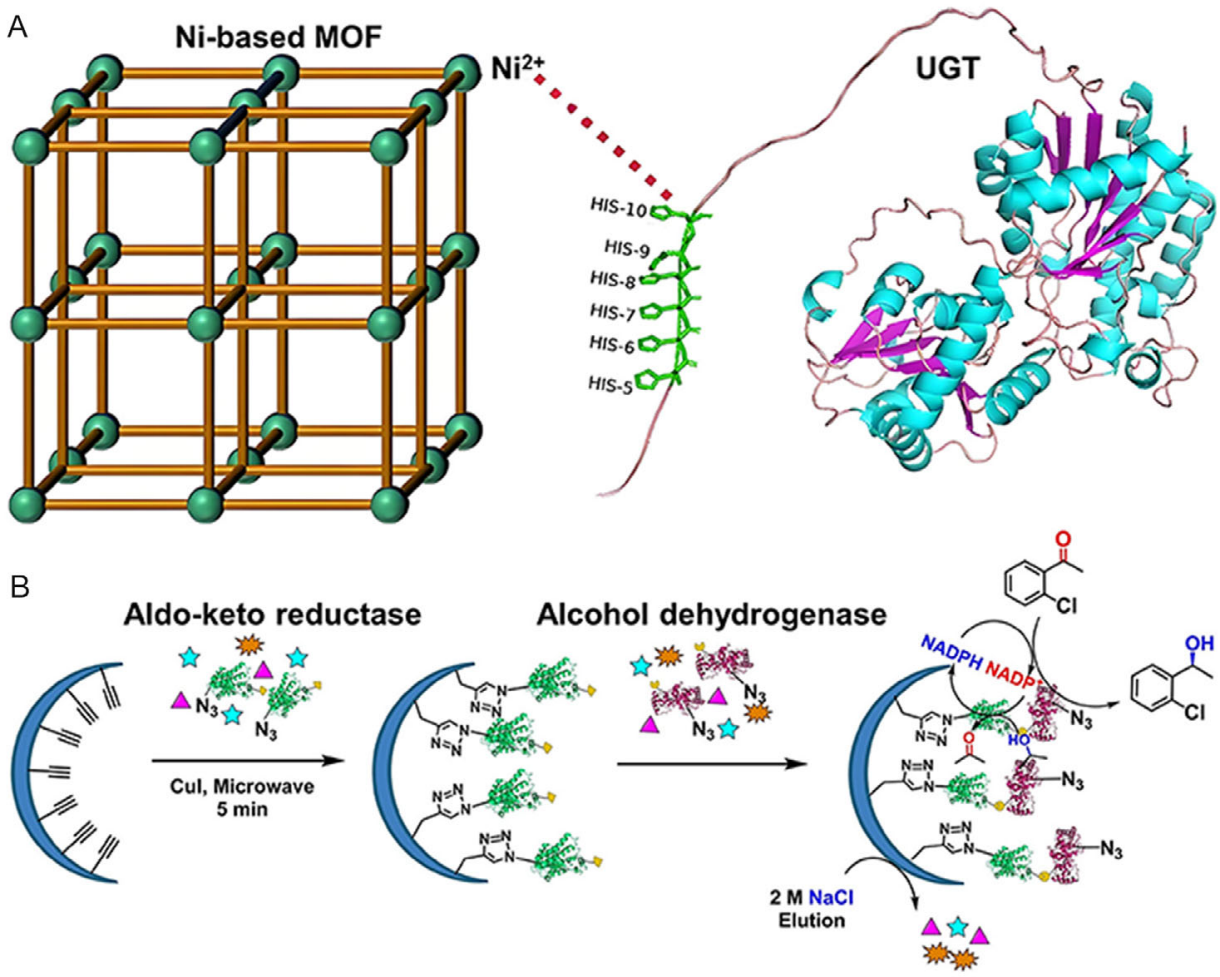
A) Schematic illustration of one‐step purification and immobilization of UDP‐glucosyltransferases with Zn‐Ni MOF. Reproduced with permission.^[^ ^35^ ^]^ Copyright 2024, American Chemical Society. B) Schematic illustration of ordered immobilization of dual‐enzyme by click chemistry. Reproduced with permission.^[^ ^40^ ^]^ Copyright 2024, Wiley.

Surface attachment refers to anchoring the enzyme to the surface of a presynthesized MOF. Based on the nature of the interactions, surface attachment of the enzyme can be classified into two main types: physical adsorption and covalent attachment. Physical adsorption is the simplest and most straightforward approach for preparing MOF‐enzyme hybrids, relying on noncovalent forces such as van der Waals interactions, hydrophobic effects, π–π stacking, electrostatic interactions, and coordination bonds.^[^ ^32^ ^]^ Owing to its straightforward implementation and efficient utilization of the MOF surface, this immobilization approach has been extensively employed in the field of electrochemical sensing. By employing MIL‐100(Fe) Materials of Institute Lavoisier (MIL) as the immobilization matrix for glucose oxidase (GOx), the resulting bioelectrode demonstrates outstanding electrocatalytic efficiency, featuring a low detection limit of 5 μM and a rapid response time of less than 5 s.^[^ ^33^ ^]^ In addition to post‐synthetic modification of MOFs to introduce functional groups for enzyme adsorption, MOFs themselves possess numerous coordination sites that can directly interact with amino acid residues, thereby facilitating enzyme immobilization.^[^ ^34^ ^]^ By leveraging coordination interactions between His‐tagged UDP‐glucosyltransferases and Zn‐Ni MOF (**Figure** **3**A), simultaneous one‐step purification and immobilization directly from crude enzyme solutions was achieved. The resulting composite demonstrated excellent stability and reusability, retaining ≈76% of its initial catalytic activity after seven consecutive cycles.^[^ ^35^ ^]^

Due to the reversibility of these noncovalent bonds, physical interactions alone are insufficient to prevent enzymes from leakage.^[^ ^36^ ^]^ In applications such as biocatalysis, where enzyme recyclability is a critical requirement, the need for stronger enzyme anchoring on the MOF surface becomes more pronounced.^[^ ^37^ ^]^ In these instances, covalent bonding is employed to achieve more stable and durable enzyme immobilization.^[^ ^38^ ^]^ Leveraging the tunable amino groups of MIL‐101‐Fe‐NH_2_, the model antigen ovalbumin was covalently conjugated to the MOF surface via disulfide bonds, enabling reduction‐responsive antigen delivery.^[^ ^39^ ^]^ Click chemistry represents an additional strategy for establishing covalent connections. Luo and coworkers successfully integrated noncanonical amino acid incorporation with click chemistry to facilitate the sequential immobilization of alcohol dehydrogenase and aldo‐keto reductase (Figure 3B), ultimately realizing effective cascade catalysis with 1.77 times higher efficacy than that of disorderly co‐immobilized enzymes.^[^ ^40^ ^]^ Overall, covalent attachment of proteins to MOF surfaces has proven to be a powerful strategy for constructing a wide range of novel protein‐on‐MOF biocomposites.

Overall, in surface attachment strategies, enzymes are immobilized onto pre‐synthesized MOFs, allowing independent preparation of MOFs and enzymes, eliminating mutual interference during synthesis. This approach is broadly compatible with a wide variety of MOFs and enzymes. Additionally, the high specific surface area of MOFs ensures native conformation of immobilized enzymes. However, surface attachment is often faced with issues such as enzyme leakage and insufficient protection. Other immobilization strategies offer distinct advantages and limitations, as outlined in **Table** **1**.

**Table 1:** Comparison of enzyme immobilization methods based on MOFs, and the corresponding main advantages and drawbacks.

	Free enzyme	Surface attachment	Pore infiltration	Encapsulation
Advantages	Ready to use	Availability for large numbers of MOFs	No effects on immobilization during the synthesis of MOFs	Facile one‐pot synthesis
Drawbacks	Fragility	Leakage of the enzyme	Limited number of mesoporous MOF	Mass transfer restriction
Protection	None	Low	Medium	High
Mass transfer	High	High	Medium	Low
Typical applications	Antibody, flag	Detection, toxin degradation	Biocatalysis, delivery	Therapy, biocatalysis
Preparation	Purification	Single step	Multistep	Limited to particular MOFs

### 2.2 Pore Infiltration

Pore infiltration means introducing an enzyme within the pore network of a presynthesized MOF. Enzyme infiltration into preformed MOF requires careful design, particularly the selection of frameworks with sufficiently large pores to accommodate the enzyme, facilitate substrate access, and release product.^[^ ^41^ ^]^ The required pore size depends on the adsorption mechanism, and reversible adsorption allows for smaller pores.^[^ ^42^ ^]^ To minimize enzyme leaching and preserve native conformation, stabilizing interactions provided by tailored organic functional groups are essential.^[^ ^43^ ^]^ Advanced designs employ hierarchical pore structures, positioning enzymes within larger pores while enabling efficient diffusion of cofactors, substrates, and products through smaller channels.^[^ ^44^ ^]^ Such systems are well‐suited for multienzyme cascades and hybrid catalysis. Moreover, the framework must remain stable under operational conditions,^[^ ^45^ ^]^ which are often distinct from optimal biological environments and are generally compatible with sequential synthesis and infiltration processes. Hierarchically porous MOFs were developed through controlled structural etching, creating enlarged pores that accommodate both NAD^+^ cofactor and l‐glutamate dehydrogenase for enhancing reactant and intermediate diffusion. This design achieved up to a 16‐fold increase in enzymatic activity compared to unetched MOFs.^[^ ^46^ ^]^ By accommodating esterase within the pores of NU‐1000 and integrating the resulting MOF‐enzyme hybrid into a high‐performance liquid chromatography column as a continuous‐flow bioreactor, a high space‐time yield of 1432 g L^−1^ h^−1^ and enhanced enzyme stability by ≈30‐fold compared to the free enzyme were achieved.^[^ ^47^ ^]^

**Figure 4:**
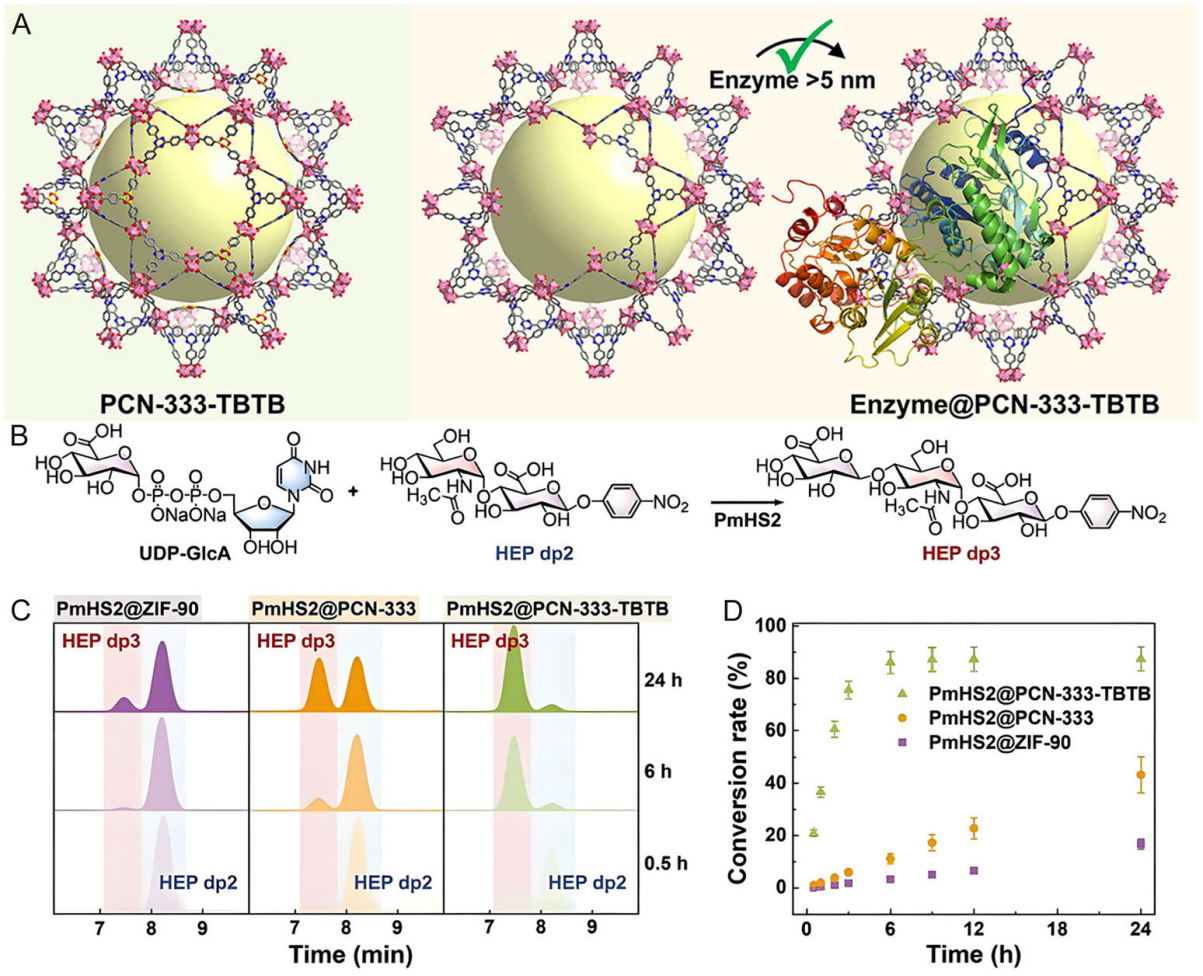
A) Schematic illustration of ligand exchange‐mediated enzyme loading process of PCN‐333‐TBTB (PCN, porous coordination network). B) The synthesis of HEP dp3 catalyzed by *Pasteurella multocida* heparosan synthase 2 (PmHS2). C) HPLC monitor and D) the conversion rate of reactions catalyzed by PmHS2‐encapsulated PCN‐333‐TBTB, PCN‐333, and ZIF‐90 (ZIF, zeolitic imidazolate framework). Reproduced with permission.^[^ ^49^ ^]^ Copyright 2024, Wiley.

Offering tunable pore sizes, hierarchical architectures, and customizable chemical environments tailored to the size and surface properties of enzymes, mesoporous MOFs are promising hosts for enzyme infiltration.^[^ ^48^ ^]^ Their crystallinity further ensures precise pore size control. For example, dynamic boroxine linkers were introduced into mesoporous PCN‐333‐TBTB to create defects that enlarge pore sizes, allowing the infiltration of enzymes exceeding the intrinsic pore dimensions (**Figure** **4**A). The resulting glycoenzyme‐loaded PCN‐333‐TBTB enabled the efficient synthesis of thirteen complex oligosaccharides and polysaccharides (Figure 4B), exhibiting high catalytic activity and improved enzyme stability (Figure 4C and D).^[^ ^49^ ^]^ These initial studies established a foundation for enzyme stabilization through pore infiltration into mesoporous MOFs. However, deeper investigations into infiltration mechanisms and stabilization effects are still highly needed.^[^ ^50^, ^51^ ^]^ Most mesoporous MOFs rely on large aromatic linkers to construct the mesoporous structure, and the side functional groups are hardly explored to maximize the pore size.^[^ ^52^ ^]^ Modifications to enhance MOF‐enzyme interactions, such as hydrophilicity or hydrogen bonding, deserve consideration. Additionally, advanced investigation of adsorption behavior within modified MOF environments should be conducted, which can furnish a fundamental understanding of MOF‐enzyme interface chemistry.

### 2.3 Encapsulation

Encapsulation involves synthesizing MOFs in the presence of enzymes, resulting in MOF‐enzyme hybrids where the enzyme is encased within the MOFs’ structures. Typically, enzyme infiltration into MOFs requires relatively larger pore sizes to ensure diffusion into the pore network.^[^ ^53^ ^]^ One‐step synthesis of MOF‐enzyme hybrids by using the enzymes as the nucleus for MOFs growth can realize the de novo encapsulation of enzymes. Compared to pore infiltration, encapsulation offers the advantage of decoupling MOF pore size from enzyme dimensions.^[^ ^54^ ^]^ Encapsulated enzymes generally exhibit superior recyclability compared to surface‐attached enzymes, since the MOF shell minimizes enzyme loss.^[^ ^55^ ^]^ In contrast, surface‐attached enzymes are prone to desorption during washing and reuse due to random orientation and progressive leaching, which can be mitigated by covalent attachment.^[^ ^56^ ^]^ However, dynamic processes such as linker exchange still induce enzyme release.^[^ ^57^ ^]^ Encapsulation also enables straightforward one‐pot synthesis of MOF‐enzyme composites.^[^ ^58^ ^]^ Meanwhile, one‐pot encapsulation can restrict substrate and cofactor access, and the approach is limited to MOFs that can be synthesized under mild and biologically compatible conditions.^[^ ^59^ ^]^ Thus, the key is the enhancement of the selective permeability of MOF pores.

**Figure 5:**
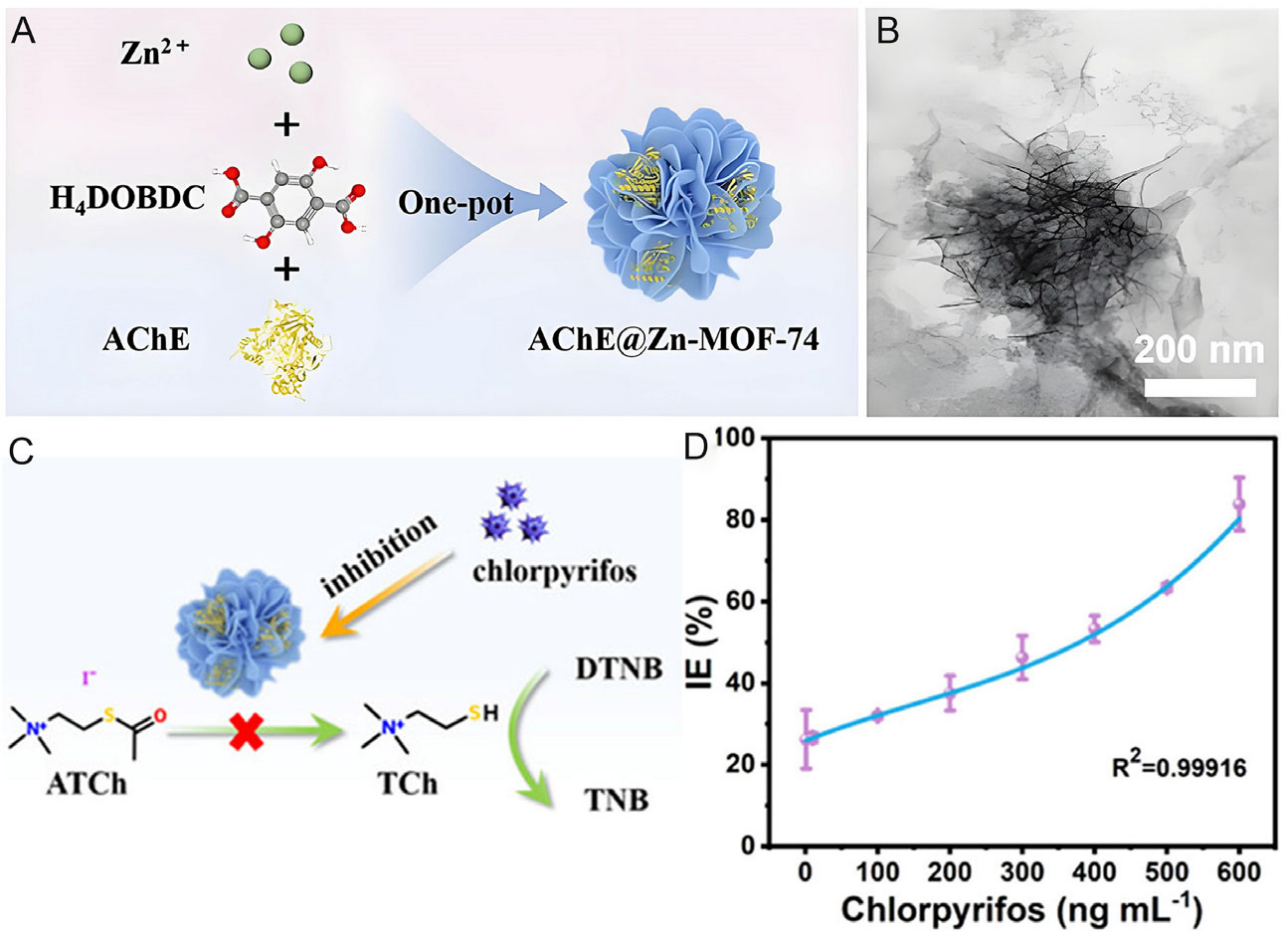
A) Synthesis process of Zn‐MOF‐74 with the nanoflower architecture. B) Transmission electron microscope image of Zn‐MOF‐74. C) Colorimetric sensing platform for detection of chlorpyrifos based on AChE@Zn‐MOF‐74. D) Standard curve of inhibition efficiency (IE) and concentrations of chlorpyrifos. Reproduced with permission.^[^ ^60^ ^]^ Copyright 2024, Wiley.

To boost the mass transfer process, Zn‐MOF‐74 with the nanoflower architecture was prepared (**Figure** **5**A,B). Benefiting from the MOF‐provided hydrophilic microenvironment, encapsulated acetylcholinesterase maintained 90% activity. The composite‐based biosensor achieved sensitive detection of chlorpyrifos with a detection limit of 1.0 ng mL^−1^ (Figure 5C,D).^[^ ^60^ ^]^ By developing hollow NH_2_‐MIL‐88(Fe) nanospheres with interfacial defects to enhance reactant diffusion, a 2.6‐fold increase in laccase catalytic efficiency compared to the free enzyme was achieved.^[^ ^61^ ^]^ Similarly, site‐selective epitaxial growth was employed to fabricate MOFs with mesopores and defect sites, effectively enlarging pore size to facilitate enhanced molecular transport.^[^ ^62^ ^]^

In addition to structural engineering of MOFs, enhancing the intrinsic activity of encapsulated enzymes offers an effective strategy to mitigate mass transfer limitations. Chen et al. designed a MOF incorporating metal ion activators as building blocks, resulting in a significant increase in the enzymatic activity of up to 251%.^[^ ^63^ ^]^ By co‐encapsulating GOx with ultrasmall arginine‐derived carbon dots into ZIF‐8, enhanced enzyme stability and significantly improved electrochemical sensing performance compared to the carbon dot‐free control were achieved.^[^ ^64^ ^]^ Esterase exhibited superior catalytic activity when encapsulated in a phase‐pure metal azolate framework compared to ZIF‐8 and ZIF‐90, which can be attributed to the stabilized enzyme's open‐lid active conformation in the hydrophobic microenvironment of frameworks.^[^ ^65^ ^]^

Although encapsulation offers superior protection and recyclability compared to infiltration and surface attachment strategies, the approach faces several limitations. First, the preparation of MOF‐enzyme composites must be mild and biocompatible. Second, reaction rates are often limited by mass transfer barriers. Third, de novo encapsulation strategies are largely restricted to a narrow range of MOFs, particularly ZIFs, due to their compatibility with aqueous conditions.^[^ ^66^, ^67^ ^]^ However, ZIFs suffer from instability in acidic environments and phosphate.^[^ ^68^ ^]^ Additionally, ZIFs possess small pore apertures, which further restrict substrate diffusion.^[^ ^69^ ^]^

From a synthetic standpoint, enzyme immobilization strategies using MOFs can be broadly categorized into one‐pot synthesis and postsynthetic strategies. De novo encapsulation is a representative one‐pot strategy, wherein enzymes are incorporated during the in situ formation of MOFs. However, it is important to note that one‐pot methods cannot ensure the successful incorporation of enzymes. In some cases, the MOFs crystallize without integrating the enzyme into their framework, and immobilize enzymes by surface adsorption. Consequently, it is essential to characterize whether the enzyme is truly encapsulated within the MOF interior. Furthermore, enzymes within MOFs may compromise their native structure and activity due to the microenvironment of MOFs. Surface attachment and pore infiltration can be classified as a post‐synthetic strategy, in which presynthesized MOFs are subsequently combined with enzymes. This decoupled approach allows both components to be synthesized independently, thereby preserving enzyme activity by avoiding the harsh conditions associated with MOF synthesis. Surface‐immobilized enzymes generally retain their native conformation; however, their long‐term stability may be limited due to insufficient structural protection. Although pore infiltration also falls under post‐synthetic modification, it requires MOFs with suitably large and accessible pores to accommodate enzyme diffusion into the internal structure. Therefore, the selection of an appropriate immobilization strategy should be guided by the specific requirements of the intended application. Moreover, advances in precise MOF design provide promising avenues to address these limitations. The following sections will explore these design strategies in detail.

## 3 MOF Engineering for Improved Enzymatic Performance

As mentioned above, the performance of MOF‐enzyme hybrids is often unsatisfactory due to factors such as limited mass transfer, restricted conformational flexibility of encapsulated enzymes, and immobilization in an accompanied unfavorable microenvironment.^[^ ^70^, ^71^, ^72^ ^]^ Fortunately, the microenvironment of anchored enzymes can be improved by adjusting the surface and pore of MOFs. To overcome these challenges and enhance enzymatic performance, researchers devote themselves to the design and systematic engineering of MOF structures. This section provides a comprehensive overview of recent advancements aimed at improving the functionality of MOF for better enzymatic performance. According to the engineered region of MOFs, these strategies can be generally classified as surface modification, pore design, morphological tuning, and defect engineering.^[^ ^73^ ^]^

### 3.1 Modulating Surface Microenvironments of MOFs

When placed at a favorable microenvironment, enzymes encapsulated within MOFs can exhibit enhanced catalytic activity. As so far, researchers have explored various strategies to engineer microenvironments that can optimize enzymatic activity. One of the most common approaches is modulating surface polarity of MOFs, and the other is polymer‐based coating.

**Figure 6:**
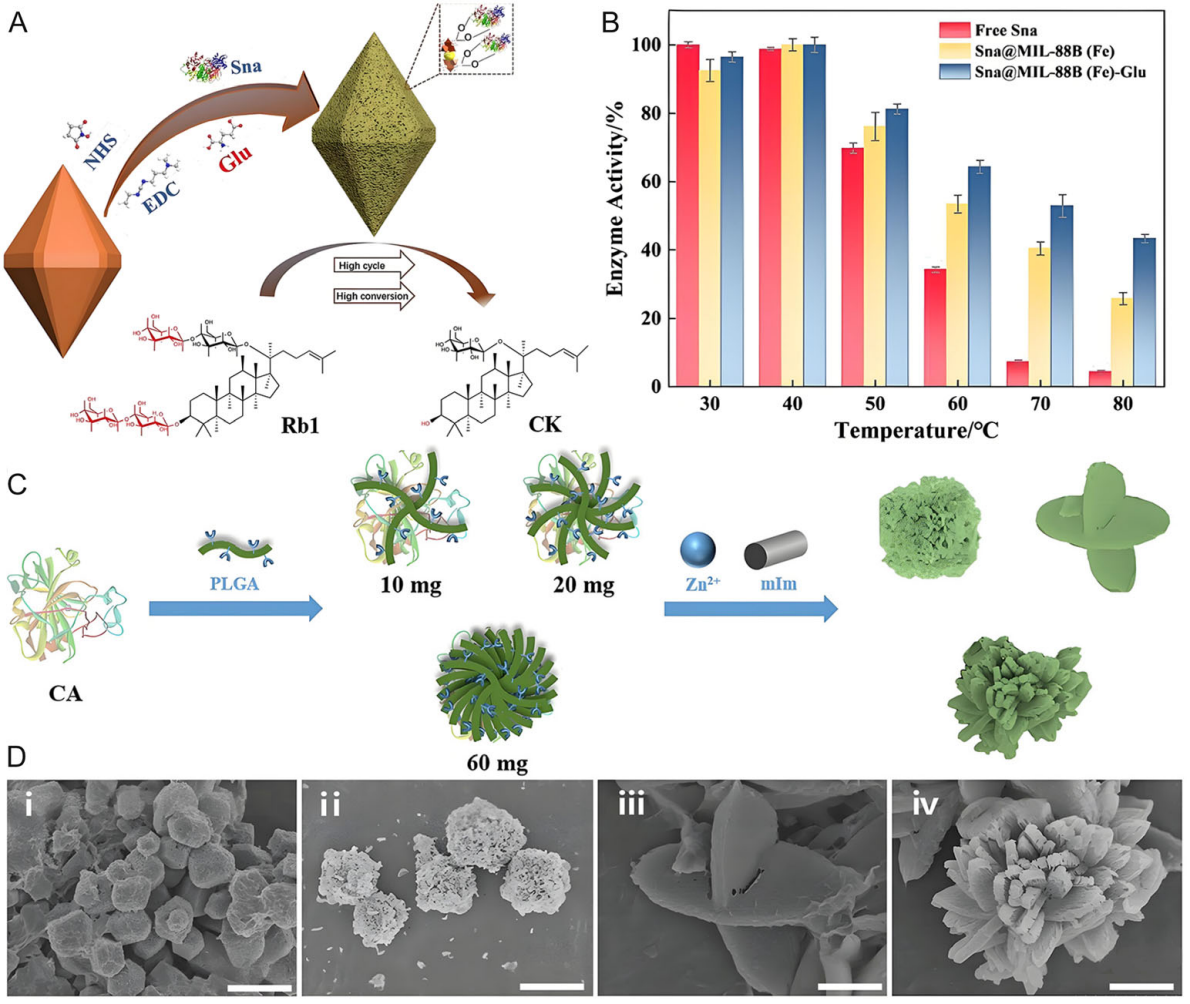
A) Schematic illustration of immobilization of Sna on glutamate modified MIL‐88B(Fe). B) Stabilities of free Sna, Sna@MIL‐88B(Fe) and Sna@MIL‐88B(Fe)‐Glu at different temperatures. Reproduced with permission.^[^ ^75^ ^]^ Copyright 2025, Elsevier. C) Schematic illustration of PLGA‐regulated preparation of CA@ZIF‐8. D) Scanning electron microscope images of CA@ZIF‐8 with PLGA i) 0 mg, ii) 10 mg, iii) 20 mg, and iv) 60 mg as regulator. Reproduced with permission.^[^ ^79^ ^]^ Copyright 2023, Springer.

For the first strategy, functional groups based MOF surface polarity modification has been shown to improve hydrophilicity, thereby promoting enzymatic activity. For example, given the hydrophilic nature of the enzymes, MOF MIL‐101(Al)‐NH_2_ was synthesized using the ligand with an amino group to contribute to surface hydrophilicity. The resulting composite exhibited high tolerance to extreme pH (1–12) and temperatures (95 °C). Besides, the MOFs protection endows the composite with exceptional stability and reusability over 15 cycles.^[^ ^74^ ^]^ Similarly, glutamate was modified on MIL‐88B(Fe) to introduce carboxyl and active methylene (**Figure** **6**A), increasing the surface density of reactive groups. Immobilized snailase (Sna) retained 40% activity, while free Sna was completely denaturated at 80 °C (Figure 6B).^[^ ^75^ ^]^ Although enzymes are generally more stable in aqueous phases, introducing hydrophilic groups onto the MOF surface does not necessarily enhance enzymatic catalytic efficiency. In fact, catalytic efficiency is also influenced by conformational changes of the enzyme. For instance, the catalytic center of lipase is covered by a lid structure, and a hydrophobic environment can facilitate the opening of this lid, allowing substrates to access the active site more easily.^[^ ^76^ ^]^ Taking advantage of this concept, a series of fatty acids were conjugated to UiO‐66‐NH_2_ (UiO, University of Oslo), and lipase B from *Candida antarctica* was then immobilized on the modified MOFs, achieving 13‐fold conversion efficiency compared to free lipase B.^[^ ^77^ ^]^

The intrinsic surface polarity of some MOFs, particularly 2‐methylimidazole based MOFs, is often difficult to modify. In that case, introducing functional polymers to shield enzymes would be an alternative strategy. These polymers can coordinate with metal ions, accelerating the assembly of MOFs. Meanwhile, their high affinity for enzymes can facilitate the encapsulation of enzymes within MOFs. Thus, enzymes encapsulated in MOFs are placed in a favorable microenvironment, maintaining their native conformation and activity to the greatest extent. To enhance enzyme adsorption, MOFs are often functionalized in advance to introduce favorable surface properties. For example, by coating polydimethylsiloxane on MOF (UiO)‐66, *Aspergillus niger* lipase was immobilized via hydrophobic interactions, achieving 88% yield of biodiesel at 24 h.^[^ ^78^ ^]^ For another example, poly‐l‐glutamic acid (PLGA) was adopted to regulate the synthesis of carbonic anhydrase@ZIF‐8 hybrids (Figure 6C). PLGA stabilizes and protects the enzyme through electrophilic interactions while accelerating MOF crystallization via coordination with Zn^2+^, thereby promoting the de novo encapsulation of carbonic anhydrase within ZIF‐8. Additionally, the introduction of PLGA influences the morphology of MOFs. With increasing the PLGA content, a nanoflower‐shaped MOF is ultimately formed (Figure 6D),^[^ ^79^ ^]^ which further enhances mass transfer and improves the activity of the MOF‐enzyme hybrid. Compared to hybrid without PLGA, the PLGA‐introduced hybrids showed nearly twofolds’ enhancement in CO_2_ conversion rate.

Optimizing MOF‐enzyme interactions by tuning surface polarity of MOFs are beneficial to maintain native conformation and thereby enhancing enzymatic activity. Notably, the adjustment approaches and directions cannot be generalized but should be designed based on the specific properties of the enzymes and MOFs. Careful selection of MOF ligands, ligand side‐group, and modulators can maximize enzymatic activity. Additionally, the well‐designed MOF pore channels can serve as protective barriers against denaturation under harsh conditions.

### 3.2 Designing Pore Size and Volume of MOFs

In most cases, MOFs are obtained with their apertures smaller than 2 nm.^[^ ^80^ ^]^ While most enzymes exceed 2 nm in size, immobilization of enzymes in MOFs pores is significantly limited. Luckily, the tunability of MOFs metal nodes and ligands allows for precise control over pore size and volume, enabling enzymes infiltrating into engineered MOFs pores. A series of MOFs with large cavities have emerged; while large cavities facilitate enzyme infiltration and enhance mass transfer, the MOF itself serves as a protective shell, ensuring enzymatic stability and catalytic activity.^[^ ^81^, ^82^, ^83^ ^]^

**Figure 7:**
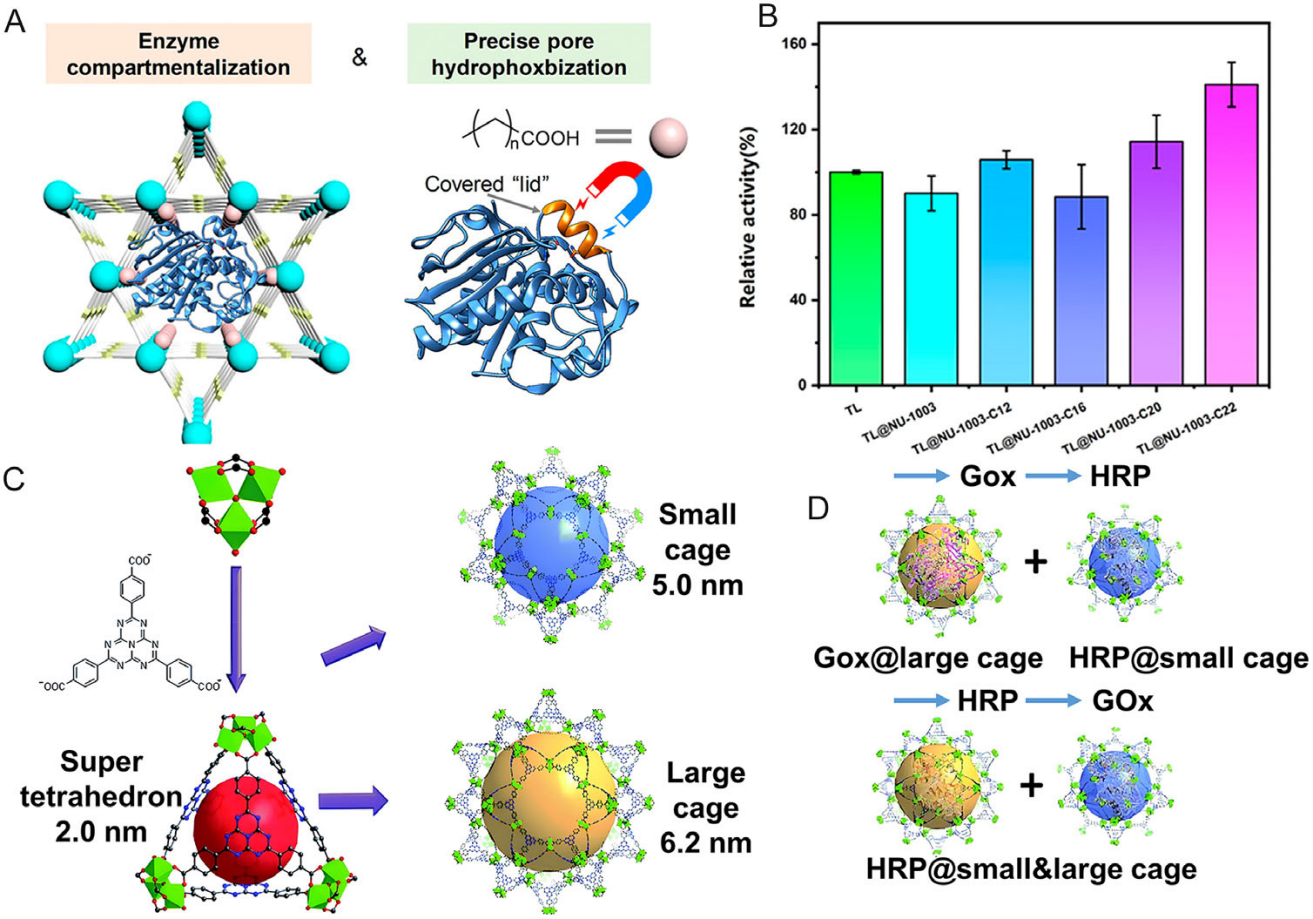
A) Schematic illustration of the pore‐hydrophobization strategy for activating lipase in fatty acid modified NU‐1003. B) Relative activity of free lipase, lipase in NU‐1003, and lipase in fatty acid modified NU‐1003. Reproduced with permission.^[^ ^85^ ^]^ Copyright 2024, American Chemical Society. C) The super tetrahedron structure of PCN‐888 and three distinct cavities constructed by the tetrahedron structure. D) Tandem immobilization of GOx and HRP. Reproduced with permission.^[^ ^87^ ^]^ Copyright 2016, Royal Society of Chemistry.

A representative strategy for expanding MOFs’ pore and aperture size through ligand design was demonstrated by Farha's group. When ligand extension proved ineffective, they synthesized with a pyrene‐based tetracarboxylate ligand, leading to the formation of MOFs NU‐1003, which features hexagonal pores up to 44 Å. Organophosphorus acid anhydrolase immobilized within NU‐1003 presented enhanced catalytic efficiency compared to its free form.^[^ ^84^ ^]^ Another representative pore engineering strategy was reported by Ouyang's group. By modifying the mesopores of MOF NU‐1003 with tunable fatty acid chains, hydrophobic environments were created to induce the open conformation of lipase via interfacial activation (**Figure** **7**A). This design enhanced ester hydrolysis activity by 1.57‐ and 2.46‐fold compared to native lipase (Figure 7B).^[^ ^85^ ^]^ Zhou's group developed a series of stable MOFs with precisely designed ultra‐large mesoporous cages, serving as single‐molecule traps for enzyme. The tailored ultra‐large cavities of PCN‐333 fulfill key criteria for an effective enzyme support. All three selected enzymes exhibited high loading capacity, enhanced catalytic efficiency, and improved recyclability.^[^ ^86^ ^]^ Zhou's group further designed PCN‐888 MOFs to co‐immobilize GOx and horseradish peroxidase (HRP) as a tandem nanoreactor. PCN‐888 features three distinct cavity types: the largest (6.2 nm) accommodates a single GOx molecule, the intermediate (5.0 nm) selectively hosts one HRP molecule (Figure 7C and D),^[^ ^87^ ^]^ while the smallest (2.0 nm) is insufficient for either enzyme and serves as a substrate diffusion channel. This spatially confined nanoreactor exhibits excellent catalytic activity and stability with minimal enzyme leaching over multiple catalytic cycles.

The immobilization of a wide range of valuable enzymes has extensively explored via pore engineer, including catalase, lipase, cutinase, anhydrolase and dehydrogenase.^[^ ^88^, ^89^ ^]^ Given that enzyme immobilization and the relatively harsh conditions of MOF synthesis are temporally and spatially separated in pore engineer strategy, further refinement of this approach is feasible. Additionally, advanced techniques such as microfluidics and electrospray have demonstrated significant advantages in MOF‐based enzyme immobilization.^[^ ^90^, ^91^ ^]^ However, the limited mass transfer of substrates may constrain the efficiency of MOF‐enzyme hybrid with enzymes trapped in pores. Precise chemical modulation with MOF pores is still highly desired for expanding advanced applications.

### 3.3 Tuning Morphology of MOFs

The performance of MOFs is primarily dictated by factors such as shape, size, and porosity. By strategically selecting metal ions and ligands and fine‐tuning synthesis parameters, MOFs with tailored morphologies and optimized properties can be engineered.^[^ ^92^ ^]^ For example, nanoflower‐shaped MOFs can facilitate efficient mass transfer, and such structures can be obtained through in situ growth or postsynthetic modifications. Extensive studies on MOF crystallization have provided critical insights into the precise control of synthesis conditions to achieve targeted morphologies, porosity, crystallinity, and even amorphous structures.^[^ ^93^ ^]^

**Figure 8:**
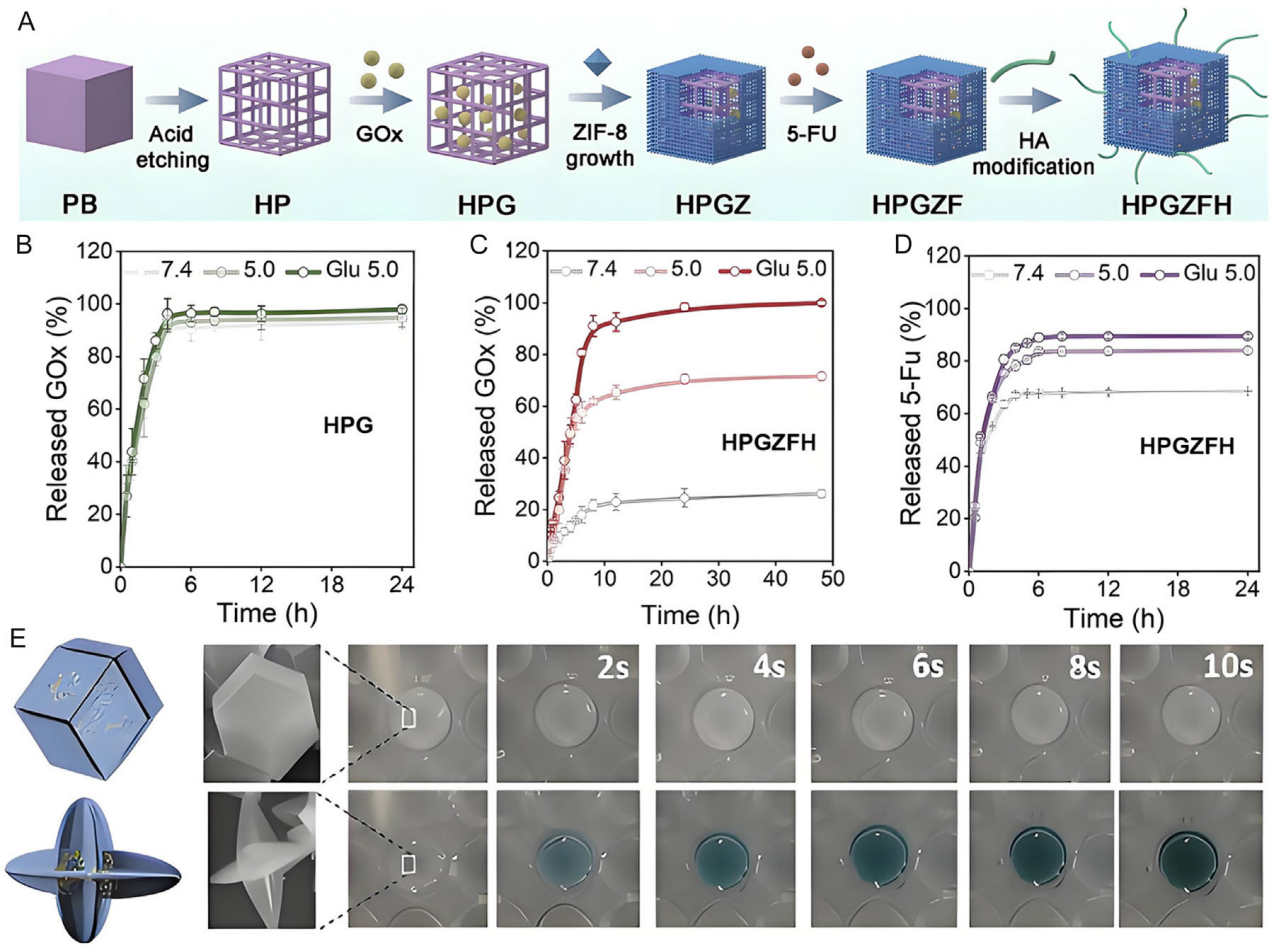
A) Synthetic route of Prussian blue based porous MOF heterostructure with well‐organized hierarchical pores. GOx release profiles of B) HPG and C) HPGZFH under different pHs in glucose solution. D) 5‐FU release profiles of HPGZFH. Reproduced with permission.^[^ ^94^ ^]^ Copyright 2024, American Chemical Society. E) The visual catalytic process of glucose biosensor (top, 3D microporous MOF; bottom, 2D mesoporous layer MOF). Reproduced with permission.^[^ ^106^ ^]^ Copyright 2020, Wiley.

Typically, hierarchically porous MOFs which are beneficial to mass transfer can be obtained by coprecipitation and template‐based strategy, and then enzymes can be immobilized by surface attachment. One of these studies was reported by Chen's group. By employing an epitaxial growth strategy, a microporous ZIF‐8 shell was introduced onto a hollow Prussian blue core, resulting in a porous MOFs heterostructure with well‐organized hierarchical pores. This structure enables the spatially controlled loading of the GOx and the small‐molecule drug 5‐fluorouracil within distinct pores (**Figure** **8**A). The ZIF‐8 layer effectively prevented GOx from leakage and served as an acidity‐specific switch. Under acidic tumor microenvironments, the structure can be degraded and gradually release 5‐fluorouracil (Figure 8B–D), achieving synergistically therapeutic efficacy.^[^ ^94^ ^]^ A morphological tuning strategy was reported which incorporated dodecanoic acid as a modulator during the synthesis of PCN‐224(Fe). The subsequent removal of dodecanoic acid generated large cavities, enabling the high‐capacity and stable immobilization of glucose oxidase. Given the intrinsic peroxidase‐mimicking activity of PCN‐224(Fe), the resulting composite efficiently catalyzed the cascade reaction between glucose and 2,2′‐azinobis‐(3‐ethylbenzthiazoline‐6‐sulfonate), demonstrating its potential as a glucose detection platform.^[^ ^95^ ^]^ Despite the remarkable properties of abovementioned MOFs, enzyme immobilization onto presynthesized MOFs is limited by complex synthesis protocols required to achieve MOFs with tailored pore size and volume.^[^ ^96^ ^]^ Chen's group demonstrated that the in situ formation of flower‐shaped ZIFs enables efficient enzyme de novo encapsulation.^[^ ^97^ ^]^ Because of established coordinative interactions between enzymes and Zn center, a symbiotic stabilization effect between the enzymes and ZIFs was achieved. The immobilized catalase exhibited a 45% enhancement in catalytic activity compared to its free counterpart.

Studies have highlighted the significant influence of morphology on the overall performance of MOFs, particularly in terms of surface area and mass transfer.^[^ ^98^, ^99^ ^]^ Consequently, various strategies have been developed to regulate morphology of MOFs. For instance, the addition of regulators and postsynthetic chemical etching have been adopted to modulate particle shape, size, and porosity of MOFs.^[^ ^100^, ^101^ ^]^ Notably, introduction of enzymes can directly induce MOF crystallization through a biomimetic mineralization process.^[^ ^102^, ^103^ ^]^ While these strategies provide valuable insights into morphological control, the key factors governing MOF morphology remain insufficiently understood and deeper research is still highly needed.

### 3.4 Defect Engineering of MOFs

Defect‐engineered MOFs can significantly improve enzyme immobilization by enhancing substrate diffusion and increasing enzyme loading due to the introduction of mesoporous structures.^[^ ^104^ ^]^ Defect engineering is one of the most effective method for precise modulation of MOFs properties. Beyond structural properties such as pore size, pore volume, and surface area, functional attributes including binding sites, hydrophobicity, and coordination environment can also be precisely tailored. Compared to conventional MOFs, integrating enzymes into defect‐engineered MOFs offers distinct advantages. The presence of relative larger channels facilitates the encapsulation of enzymes, and sufficient porosity allows efficient mass transfer, thereby promoting enzymatic activity.^[^ ^105^ ^]^

As discussed earlier, PLGA plays a crucial role in modulating the morphology of MOFs and enhancing enzyme encapsulation. Additionally, excess PLGA chains can competitively coordinate with metal ions via carboxyl groups, inducing defect structures and driving morphological evolution. Leveraging this strategy, Ouyang's group synthesized mesoporous spindle‐shaped MOF architectures characterized by shortened diffusion pathways and enlarged pore channels. When GOx and BSA‐scaffolded gold nanoclusters were co‐immobilized within the MOFs, a highly sensitive glucose biosensor was developed (Figure 8E), exhibiting a detection range of 0.1–1.0 mM glucose.^[^ ^106^ ^]^ The 3D MOF‐enzyme hybrids displayed low activity due to the hindered diffusion of the substrates in 3D microporous architecture. Utilizing a modulator‐induced defect formation strategy,^[^ ^107^ ^]^ hierarchically porous MIL‐53 was synthesized, in which defect‐induced mesopores significantly enhanced its capacity for enzyme immobilization. The co‐immobilized CMP‐sialic acid synthase and α‐2,6‐sialyltransferase exhibited superior catalytic efficiency, as well as enhanced thermal, pH, and storage stability compared to their free counterparts. Notably, the 6′‐Sialyllactose yield remained above 80% even after 13 cycles of reuse.^[^ ^108^ ^]^

**Figure 9:**
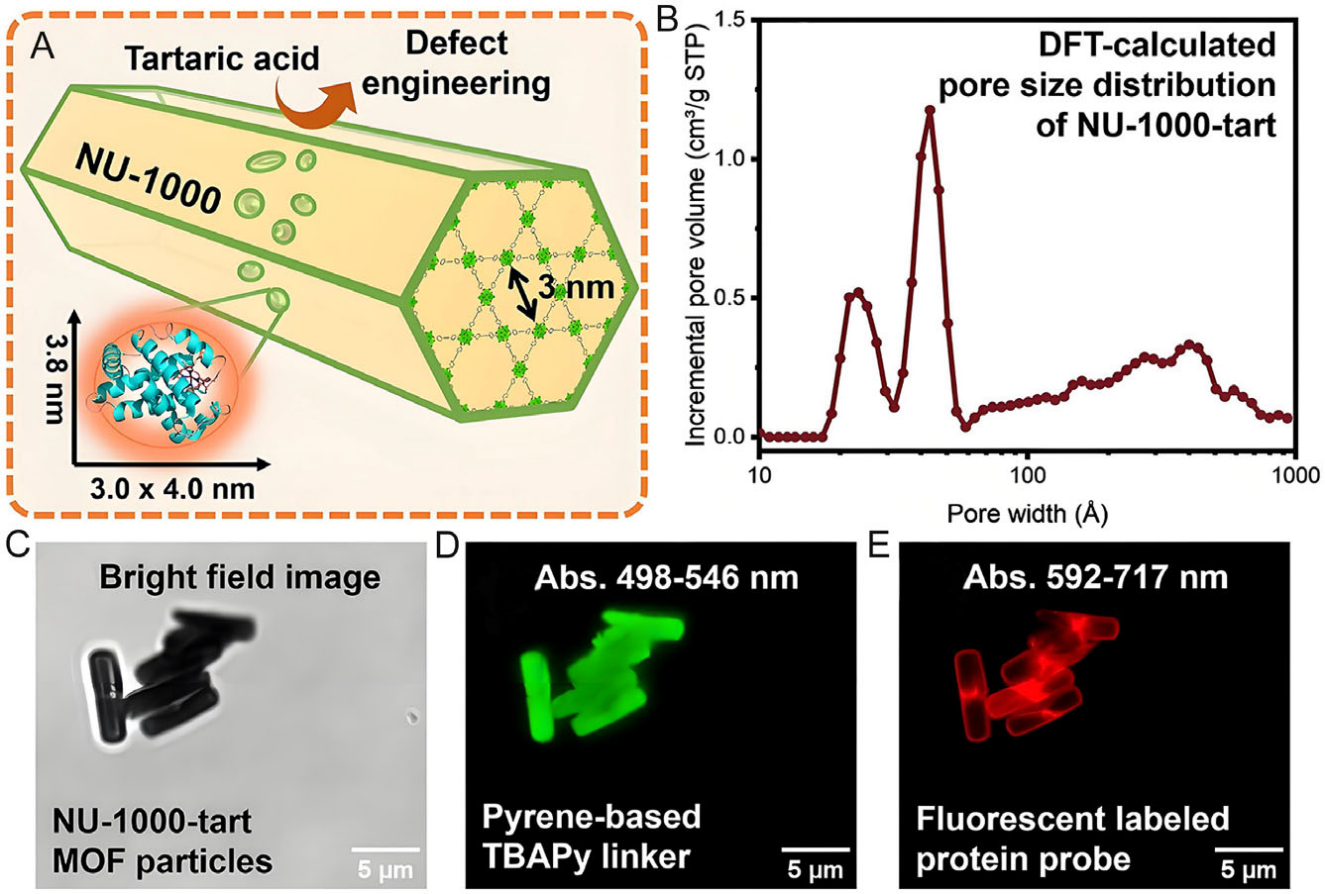
A) Schematic of the visualization of defects on NU‐1000‐tart with fluorescently labeled hemoglobin. B) Pore size distribution of NU‐1000. Confocal images of NU‐1000‐tart under C) bright field, D) green channel, and E) red channel. Reproduced with permission.^[^ ^109^ ^]^ Copyright 2024, American Chemical Society.

To characterize macroporous defects in MOFs, Farha's group employed fluorescently labeled hemoglobin as a size‐selective probe (3.0 × 3.8 × 4.0 nm) (**Figure** **9**A). Given that its dimensions exceed the crystallographic pores of NU‐1000‐tart (3 nm) (Figure 9B), this probe enabled the visualization of structural defects within the framework. Consequently, confocal microscopy revealed defect structures ranging from 10 to 100 nm in size (Figure 9C–E), which were previously undetectable by conventional techniques such as nitrogen sorption isotherms or PXRD analysis.^[^ ^109^ ^]^

Defect engineering of MOFs for enzyme immobilization has emerged as a rapidly advancing field. Structural defects generate coordinatively unsaturated metal sites and hierarchical pores within MOFs, thereby enhancing mass transfer. The resulting defect‐engineered MOFs can improve physicochemical properties that contribute to catalytic activity and stability of immobilized enzymes. Despite its remarkable progress, challenges such as identifying and regulating defects remain to be addressed. Additionally, defect engineering of MOF can lead to a decrease in the stability of MOF. Thus, balance engineering defect and structural stability is also need to be explored in the future.

## 4 MOF‐Enzyme Hybrids in Biological Applications

Enzyme immobilization on MOFs improves enzyme stability and activity under various conditions, enabling accurate biosensing, stimuli‐responsive drug delivery, and facilitated biocatalysis.^[^ ^110^ ^]^ That means, MOFs can be constructed as the host for biocatalytic reactions, serving as multifunctional platform that meets our needs. As so far, researchers have explored a wide range of enzymes with diverse catalytic functions.^[^ ^111^, ^112^ ^]^ Although different strategies may target similar goals, minimum variations in enzymes and MOFs can lead to distinct outcomes which should be carefully considered.^[^ ^113^ ^]^ An overview of MOFs based enzyme immobilization for biological applications was presented in **Table** **2** as followed.

**Table 2:** A summary of biological applications of various MOF‐enzyme hybrids.

MOF	Enzyme	Types of immobilization/optimization	Performance	Ref
PCN‐222	Cytochrome c	Pore infiltration/mesoporous MOFs	H_2_O_2_ determination, 3–4‐fold improvement in peroxidase‐mimicking activity	[164]
ZIF‐90	HRP	Encapsulation/variant gating effect	Detection of human IgG with detection limit of 100 fg mL^−1^	[165]
ZIF‐8	Lactate oxidase and HRP	Encapsulation/proximal coencapsulation	Lactate detection	[166]
ZIF‐8	GOx	Encapsulation/biomimetic mineralization	Glucose biosensor, anti‐interference in up to 20% serum	[167]
UiO‐66‐NH_2_	Acetylcholinesterase	Surface attachment	Organophosphorus detection	[168]
ZIF‐8	Carbonic anhydrase	Encapsulation	Liquid‐phase capture of CO_2_ with a 2.5‐fold increase in enzyme‐specific activity	[169]
ZIF‐8	L‐amino acid oxidase	Encapsulation/polyvinylidene fluoride coating	Synthesis of d‐amino acids, conversion rate (92.10%), and ee% (99.41%) of D‐Trp	[170]
ZIF‐8	Cellulase	Surface attachment/honeycombed morphology	Production of reducing sugar with a load (461.67 mg g^−1^) of cellulase	[171]
ZIF‐8	GOx and HRP	Encapsulation/porous networks	Removal of 80% Bisphenol A	[172]
UiO‐66‐NH_2_	Formate dehydrogenase	Pore infiltration/hierarchical structure	Reduction of CO_2_, 5.57 times higher than free enzyme system	[173]
UiO‐66(Zr)‐NH_2_	Laccase	Surface attachment	Degradation of 58% crystal violet	[174]
MIL101(Cr)‐X	Microperoxidase 8	Encapsulation/functionalized groups	Oxidation of thioanisole	[175]
ZIF‐8	Pepsin	Surface attachment/conformational optimization	Oxygen evolution reaction with overpotential of only 127 mV	[176]
ZIF67	Lipase A	Pore infiltration/mesoporous MOFs	Nitroacetates production >99 % ee	[177]
MIL‐53(Al)‐fum	Bovine serum albumin	Encapsulation	Encapsulation of bioentities with immobilization efficiency of 98%	[178]
ZIF‐8	Trypsin	Pore infiltration/microporous MOFs	Protection of trypsin	[179]
PW_12_@UiO‐67	MP‐11	Pore infiltration/electron sponge	Protection of MP‐11	[180]
NH_2_‐MIL101	Exendin‐4	Zwitterionic hydrogel‐coating/pH responsiveness	Oral exendin‐4 delivery with a relative pharmacological availability of 17.3%	[181]
MIL‐88A	Phytase	Encapsulation/defects adjustment	Controlled release of phytase from 10% to 99.7%	[182]
PCN‐224	GOx	Surface attachment	Starvation therapy/overcome hypoxia, killed 77% MCF‐7 cells	[183]
ZIF‐8	Hemoglobin and catalase	Encapsulation	Oxygen production and store oxygen in tumor	[184]
MOF‐808	Catalase	Surface attachment/supramolecular interactions	Oxygen production for photodynamic therapy, killed 85% cancer cells	[185]
ZIF‐zni	Superoxide dismutase	Encapsulation/biomimetic mineralization	Treatment of inflammatory bowel disease	[186]
PCN‐333	HRP	Pore infiltration/specific location in MOFs	Prodrug activation with IC_50_ of 4.2 mg L^−1^	[187]
ZIF‐8	GOx	Encapsulation/shorter spacing between cascade enzymes	Biocatalytic cascade for tumor therapy, inhibition of 81.5% 4T1 cells	[159]

### 4.1 Biosensing

Enzyme‐based biosensing has gained prominence in fields such as glucose monitoring, disease diagnostics, food inspection, and quality control.^[^ ^114^, ^115^ ^]^ Successful implementation in these fields requires rapid and accurate detection besides prolonged operational stability.^[^ ^116^ ^]^ While enzymes are inherently gifted at high catalytic efficiency and selectivity, the instability, reusability, and recyclability restrict their broader applications. MOFs have emerged as promising platforms for enzyme immobilization to overcome these limitations. The high surface area and porosity not only stabilize enzymes but also enhance the sensitivity and specificity of biosensors, enabling reliable detection of diverse biomolecules.^[^ ^117^ ^]^

**Figure 10:**
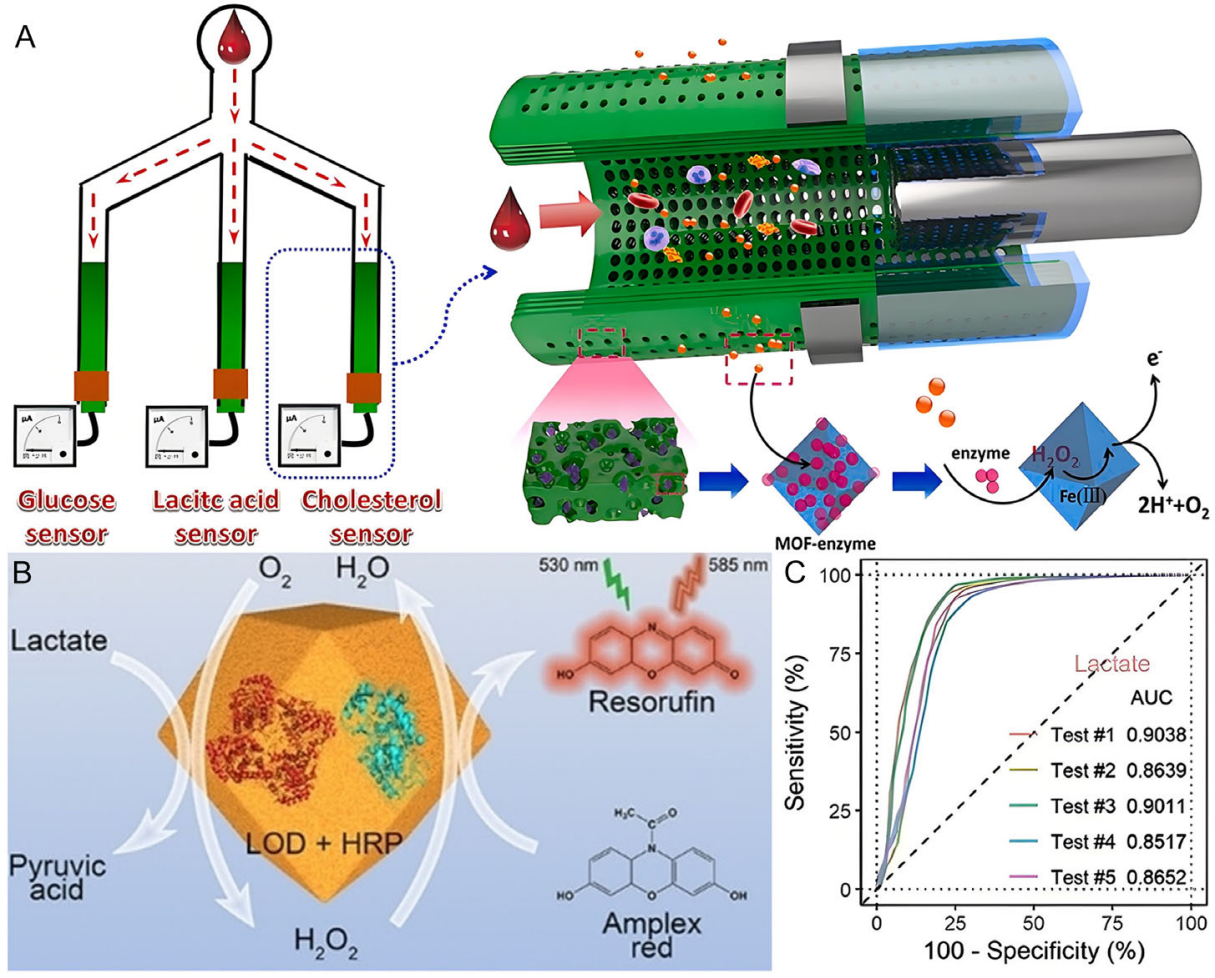
A) Schematic illustration of the MOF‐enzyme hybrid nanosystem for multiplex metabolites detection. Reproduced with permission.^[^ ^118^ ^]^ Copyright 2021, Elsevier. B) Lactate detection based on lactate oxidase and HRP loaded ZIF‐8. C) Test for the extracellular lactate level. Reproduced with permission.^[^ ^121^ ^]^ Copyright 2023, Wiley.

In addition to direct enzyme‐coupled catalytic detection, numerous approaches have been explored to improve the detection speed and sensitivity of biosensors. For example, MOF‐enzyme hybrid was incorporated into hollow fiber membranes (**Figure** **10**A), enabling efficient cascade catalysis for simultaneous detection of multiple metabolites.^[^ ^118^ ^]^ By assembling the MOF‐enzyme hybrid into 3D microporous electrodes, large surface area can be utilized for physical filtration of substrates, thereby accelerating catalytic efficiency. In another study, GOx was encapsulated within hydrophilic ZIF‐90 and integrated with Pt nanozyme, creating a cascade catalytic system that efficiently degraded 3,3′,5,5′‐tetramethylbenzidine using glucose as the substrate.^[^ ^119^ ^]^ A convenient paper‐based naked‐eye recognition was reported, where Pt nanoparticle‐loaded porous CeO_2_ and sarcosine oxidase were incorporated into a Ce‐based MOF to construct a cascade catalytic system for sarcosine detection, enabling the screening of prostate cancer patients.^[^ ^120^ ^]^ A microfluidic platform integrating lactate oxidase and HRP co‐immobilized ZIF‐8 for high‐throughput was developed, realizing real‐time monitoring of tumor cell metabolites at the single‐cell level (Figure 10B,C). This system enables precise evaluation of tumor malignancy and holds promise for screening metabolic inhibitors as anti‐metastatic agents.^[^ ^121^ ^]^ To enhance the catalytic activity of GOx and HRP co‐immobilized UiO‐66‐NH_2_, stimulus‐responsive polymer was coated on the MOF‐enzyme hybrid, forming a protective soft shell. The resulting composite exhibited an 8.9‐fold increase in catalytic activity, along with improved stability, reusability, and selectivity for visual serum glucose detection.^[^ ^122^ ^]^

Generally, future directions in this field focus on designing more robust, accurate and selective MOF‐enzyme hybrid; optimizing encapsulation strategies; and developing multienzyme systems for cascade catalysis.^[^ ^123^ ^]^ A lot of efforts such as engineering MOF surfaces to mimic natural enzyme microenvironments have been explored, thereby enhancing MOF‐enzyme interactions.^[^ ^124^, ^125^ ^]^ Notably, MOFs can mimic natural enzyme active sites through metal nodes and organic ligands, endowing them with intrinsic catalytic activity.^[^ ^126^ ^]^ Additionally, functionalization of ligands allows introduction of specific active sites onto the framework, thereby supports the assembly of multi‐enzyme cascade systems.^[^ ^127^, ^128^ ^]^ These advances aim to address current challenges and broaden the application of enzyme‐immobilized MOFs in diverse areas such as medical diagnostics and environmental monitoring.

### 4.2 Biocatalysis

Biocatalysis involves enzyme cascade reaction systems for the synthesis of diverse products, supported by advances in enzymes mining, reaction path designing, biocatalyst selection, and experimental process optimization.^[^ ^129^, ^130^ ^]^ However, ensuring enzyme stability, selectivity, and catalytic efficiency under in vitro conditions is still a significant challenge. MOF‐enzyme hybrids offer opportunities to address these limitations by enhancing enzyme stability and reusability which can be applied for biomedicine and industry.^[^ ^131^ ^]^ Though promising, maintaining consistent enzymatic performance of MOF‐enzyme hybrids remains significant challenges, including controlling enzyme orientation, minimizing diffusion limitations, and preserving catalytic efficiency in harsh environments.^[^ ^132^ ^]^ Strategies like MOF/enzyme engineering have been developed to promote the advancement of biocatalytic applications.

**Figure 11:**
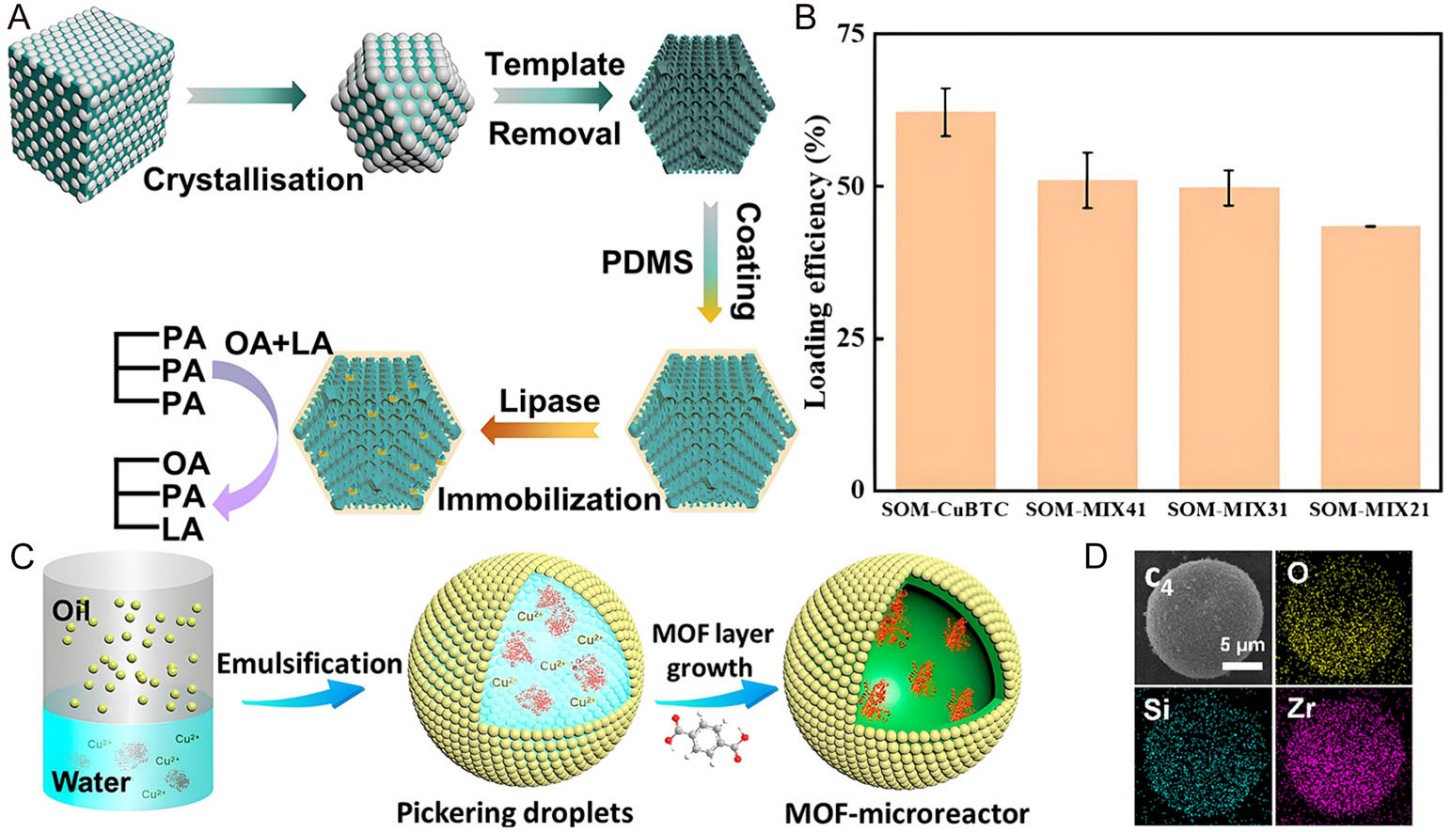
A) Synthesis of ordered macro‐microporous MOFs for immobilization of lipase. B) Loading efficiency of lipase on MOFs. Reproduced with permission.^[^ ^134^ ^]^ Copyright 2024, American Chemical Society. C) Schematic illustration of the droplet‐derived MOFs microreactors for continuous‐flow biocatalysis. D) Elemental mappings of microreactors. Reproduced with permission.^[^ ^136^ ^]^ Copyright 2021, American Chemical Society.

To improve dimensional compatibility of MOF with both lipase and substrates, mesoporous MIL‐88A was synthesized with a wide pore distribution of ≈10 nm. Compared to common MIL‐88A, the mesoporous variant exhibited superior lipase immobilization efficiency and the resulting composite effectively catalyzed the synthesis of phosphatidyl‐DHA.^[^ ^133^ ^]^ To improve mass transfer and water stability, single‐crystalline, ordered macro‐microporous dual‐ligand MOFs were developed (**Figure** **11**A), achieving enhanced lipase immobilization with a 66.9% increase in specific activity towards the synthesis of 1‐oleoyl‐2‐palmitoyl‐3‐linoleoylglycerol (Figure 11B).^[^ ^134^ ^]^ Besides, the coating of hydrophobic polydimethylsiloxane resulted in an increase of 139.1% in the catalytic specificity. To enhance lipase reusability, magnetic components were introduced during the carbonization of macroporous MIL‐88 A, yielding a magnetic composite that achieved 80% biodiesel yield and exhibited excellent reusability.^[^ ^135^ ^]^ To achieve spatially controlled reactivity and molecular sieving, an enzymatic microreactor was developed via interface‐directed synthesis, growing a MOF shell around silica emulsifier‐stabilized droplets (Figure 11C,D).^[^ ^136^ ^]^ The inner droplets provided a biomimetic microenvironment for encapsulated enzymes, while the MOF shell endowed the microreactor with size‐selective permeability. The microreactor demonstrated exceptional size selectivity, long‐term stability over 1000 h reaction, and enantioselectivity comparable to free enzymes. These studies address the enhancement of MOF‐enzyme composites from various perspectives, including catalytic activity, substrate selectivity, and environmental tolerance, thereby providing valuable insights for further optimization.

Generally, future research should prioritize the design of MOFs with enhanced structural features to improve enzyme protection, stability, and adaptability. Advancing immobilization strategies such as adopting hierarchically porous or hollow MOF, pore engineering, warrant further investigation. The development of hierarchically porous MOFs capable of supporting complex multienzyme and molecules passage holds potential for improving biocatalytic efficiency.^[^ ^137^, ^138^ ^]^ Additionally, addressing the scalability of MOF‐enzyme hybrids is critical to enable industrial translation without losing enzyme activity. Emphasis on recyclable and sustainable MOF‐based biocatalysts is essential to reduce operational costs and environmental impact, ultimately advancing the practical application of enzyme immobilization in large‐scale biocatalysis.

### 4.3 Stimulus‐Responsive Drug/Protein Delivery

The tunable chemistry of MOFs enables precise drug delivery, controllable and stimulus‐responsive release, offering significant benefits by reducing the side effects of conventional therapy.^[^ ^139^ ^]^ The potential of MOFs in controlled drug delivery systems has been increasingly recognized, with notable progress in pharmaceutical applications.^[^ ^140^ ^]^ Moreover, enzyme‐immobilized MOFs can be designed with controlled and site‐specific drug release, particularly in response to intracellular environments.^[^ ^141^, ^142^ ^]^ One canonical example is the pH‐responsive ZIFs which is often applied in tumor‐specific drug delivery.^[^ ^143^ ^]^ Another representative example was Uio‐66 MOF which was coated with clickable PEG‐phosphate ligands, achieving endogenous‐triggered drug release in the presence of high level alkaline phosphatase and enhanced therapeutic efficacy in cancer cells with elevated alkaline phosphatase expression.^[^ ^144^ ^]^ These enzyme‐responsive properties, when coupled with the functional versatility of MOFs, position them as highly promising platforms as drug delivery systems with targeted, precise, and sustained therapeutic effects.

**Figure 12:**
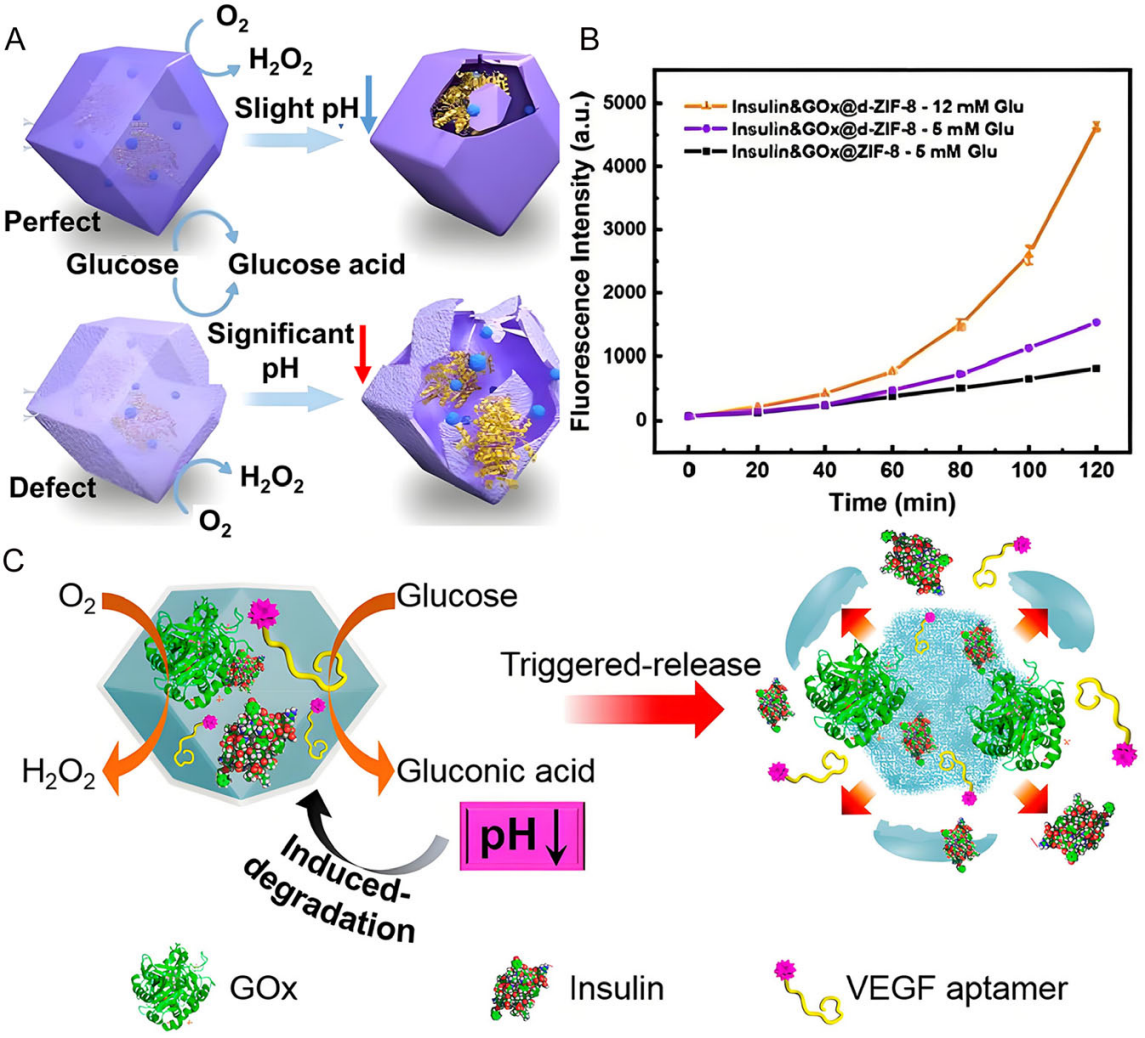
A) Schematic illustration of glucose induced smart insulin release by GOx‐embedded MOFs. B) Fluorescence intensity corresponding from FITC‐labelled insulin released from insulin&GOx@ZIF‐8 in glucose solution. Reproduced with permission.^[^ ^147^ ^]^ Copyright 2022, Elsevier. C) pH‐induced degradation of the insulin/GOx‐loaded ZIF‐8 through the GOx‐catalyzed oxidation of glucose. Reproduced with permission.^[^ ^149^ ^]^ Copyright 2018, American Chemical Society.

Due to differences of organ and tissue in gene expression, enzyme‐linked responsive drug release can serve as a customized therapeutic strategy.^[^ ^145^ ^]^ Enzymatically regulated drug delivery systems offer the useful approaches for responsive and precise therapeutic release.^[^ ^146^ ^]^ For example, co‐encapsulation of GOx and insulin within defect‐engineered MOFs enables efficient glucose‐responsive insulin release under elevated glucose concentrations (**Figure** **12**A,B).^[^ ^147^ ^]^ Other MOFs such as BTC, MAF‐7, ZIF‐8, and ZIF‐90 have also been successfully employed for effective enzyme immobilization.^[^ ^148^ ^]^ Chen et al. developed a smart glucose‐responsive carrier for the glucose‐responsive release of insulin. GOx and insulin are loaded in ZIF‐8, gluconic acid yielded by GOx shall acidize microenvironment, leading to the degradation of MOFs and the release of the loaded insulin (Figure 12C).^[^ ^149^ ^]^ Overall, immobilizing enzymes within MOFs can significantly improve their stability and therapeutic performance, underscoring the potential of MOF‐enzyme based platforms in stimulus‐responsive drug delivery.

Developing multidrug delivery systems with high drug loading efficiency, precise targeting, and stimuli responsiveness is the goal of advancing therapies.^[^ ^150^, ^151^ ^]^ MOFs have emerged as promising drug delivery platforms due to their tunable pore and easily functionalized surface.^[^ ^152^, ^153^ ^]^ For example, PCN222 achieves a high insulin loading capacity of 63 wt%, surpassing those of most polymer, liposome, and microparticle based oral insulin delivery platforms. When further functionalized with DNA to improve cellular permeability, PCN‐222 increases the cellular uptake of insulin by an order of magnitude compared to free DNA and insulin.^[^ ^154^, ^155^ ^]^ Nevertheless, challenges such as release kinetics and controllable degradation are still remaining. The concordant integration of enzymes with MOFs offers a promising strategy to overcome these limitations. Additionally, development of advanced functionalization strategies will further improve the stability, biocompatibility, and therapeutic performance of MOF‐enzyme hybrids.

### 4.4 Cancer Therapy

Conventional cancer therapies often inevitably damage healthy cells, tissues, and organs. To mitigate these adverse effects, delivery of nontoxic prodrugs that can be enzymatically converted into cytotoxic form within cancer cells has emerged as a promising approach. However, the strategy is hindered by the inherently low expression of corresponding enzymes, leading to reduced therapeutic efficacy. While delivery of exogenous enzymes into tumor cells presents a potential solution, the inherent instability of enzymes and barrier in transmembrane transport remain significant challenges. MOFs offer a compelling alternative by serving as protective, high‐capacity, and biocompatible carriers, showing potential in controlled delivery and release of therapeutic enzymes. For example, GOx was immobilized on the surface of MOFs through coordination interactions, enabling the formation of self‐stabilized, monodispersed hybrids without the need for additional surface modification, thereby facilitating efficient starvation therapy.^[^ ^156^ ^]^

**Figure 13:**
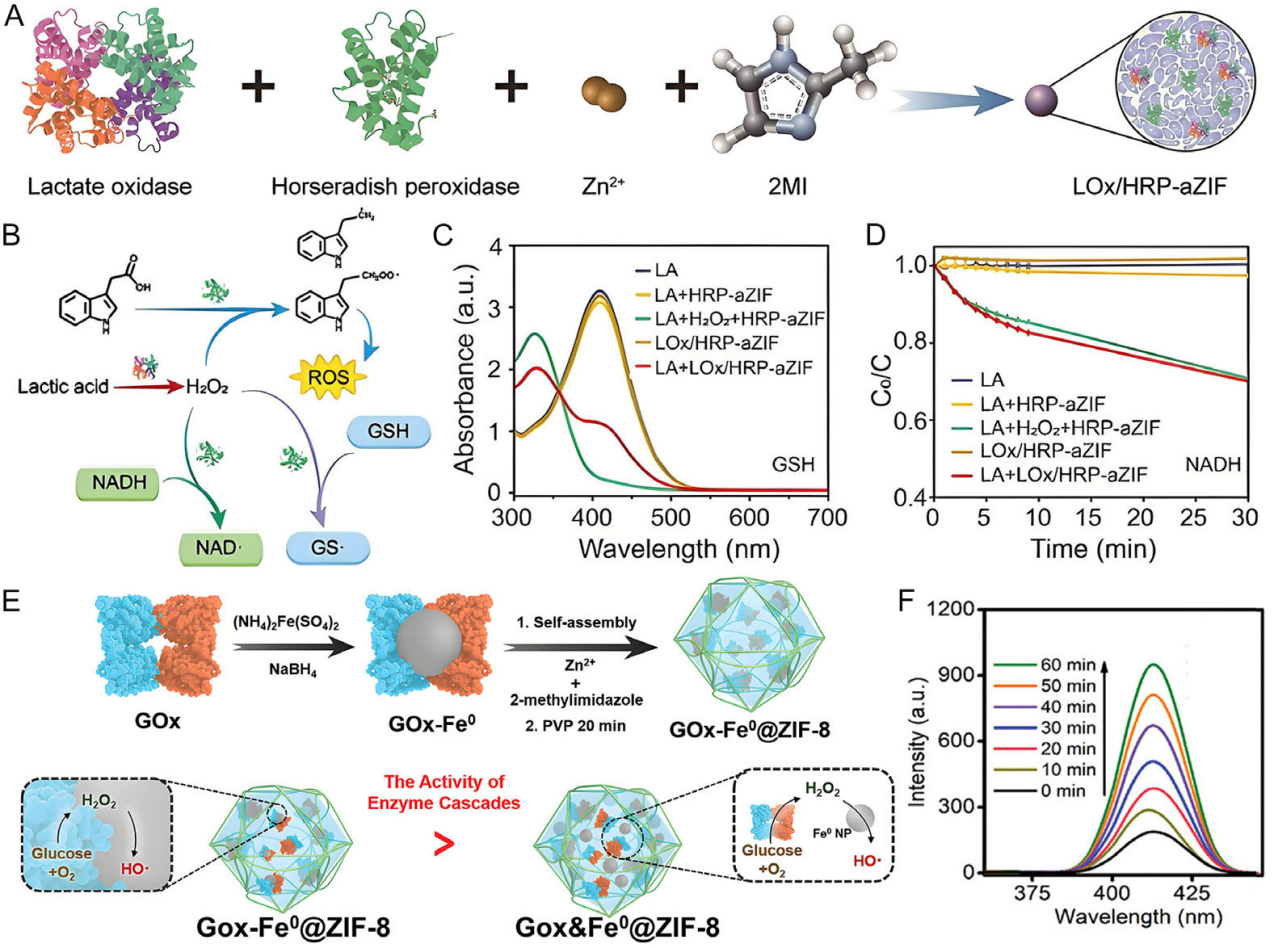
A) Therapeutic pathways of lactate oxidase, HRP, and 3‐indole‐acetic acid prodrug co‐loaded MOF. B) Schematic illustration of ROS production. C) Absorbance of 5,5′‐dithio bis(2‐nitrobenzoic acid) after different incubations. D) Time‐dependent changes of absorbance of NADH. Reproduced with permission.^[^ ^157^ ^]^ Copyright 2024, American Chemical Society. E) Schematic illustration of construction of GOx‐Fe^0^@ZIF‐8 and tumor‐specific cascade catalytic system. F) Fluorescence spectra of hydroxybenzoic acids induced by GOx‐Fe^0^@ZIF‐8. Reproduced with permission.^[^ ^159^ ^]^ Copyright 2023, Wiley.

In response to the aberrant accumulation of lactate in tumor cells, Jiang's group developed a sequential catalytic therapeutic system by co‐encapsulating lactate oxidase, HRP, and a 3‐indole‐acetic acid prodrug within an amorphous zinc‐based MOF (**Figure** **13**A). The lactate oxidase‐HRP cascade reaction produced reactive oxygen species effectively depletes endogenous glutathione, while the activation of 3‐indole‐acetic acid facilitates ferritin degradation and induces lipid peroxidation, ultimately triggering a self‐amplified ferroptotic cell death (Figure 13B–D).^[^ ^157^ ^]^ Similarly, Zhou et al. encapsulated gold nanorods, catalase, GOx, and HRP within ZIF‐8 MOFs, constructing a multifunctional trienzyme cascade system. This system efficiently depletes intratumoral glucose while simultaneously generating cytotoxic reactive oxygen species, achieving synergistically starvation and chemodynamic therapy.^[^ ^158^ ^]^ GOx and Fe^0^ nanozyme were encapsulated within pH‐responsive ZIF‐8 (Figure 13E,F),^[^ ^159^ ^]^ resulting in a tumor‐specific cascade catalytic system that selectively activates under acidic and glucose‐rich tumor microenvironment. Nanoscale Fe‐MIL‐88B‐NH_2_ was utilized as a template to fabricate urchin‐like hydrogen‐bonded organic frameworks (PFC‐1) for GOx immobilization, the resulting nanostructure facilitates a catalytic cycle wherein H_2_O_2_ decomposition generates O_2_, thereby enhancing GOx‐mediated glucose oxidation and continuous H_2_O_2_ regeneration. Furthermore, due to its extensive π‐electron conjugated system, PFC‐1 enables efficient singlet oxygen (^1^O_2_) generation under ultrasound irradiation. This multifunctional platform thus integrates chemodynamic, sonodynamic, and starvation therapy for enhanced therapeutic efficacy.^[^ ^160^ ^]^

Despite their promising potential, MOF‐based enzyme immobilization for cancer therapy suffers from several critical challenges, including maintaining structural and enzymatic stability under physiological conditions, mitigating immunogenic responses, and achieving precise control over therapeutic agent release. MOF‐induced immunogenicity may trigger adverse biological responses, necessitating rational material design to minimize immune activation. Except for struggles brought by MOFs, ensuring sustained enzymatic activity is also essential, as fluctuations of enzymatic activity will compromise therapeutic efficacy.

To achieve efficient and targeted enzyme delivery, precise engineering and rational modification of MOFs are imperative. Despite the inherent challenges and significant workload associated with such modifications, their potential benefits remain substantial. Advances in nanotechnology have created favorable conditions for optimizing enzyme release kinetics, while innovative MOF structural designs can further enhance biocompatibility and mitigate immunogenicity.

## 5 Conclusions and Future Outlook

Enzyme immobilization involves various materials and strategies, each with distinct advantages and limitations. MOFs offer notable benefits for enzyme immobilization due to their tunable composition, adjustable pore size, and easily modifiable surfaces, enabling high enzyme loading, enhanced catalytic efficiency, and improved stability under adverse conditions. Based on versatile MOFs, enzymes can be immobilized via surface attachment, pore infiltration, and encapsulation. Although encapsulation and pore infiltration can provide better protection for enzymes, but they also restrict native conformation of enzymes. Besides, pore‐size mismatch and mass transfer are always an inevitable barrier in these two strategies. In contrast, surface attachment allows flexible conformation and reduced diffusion limitations. To address this inherent trade‐off, researchers have developed a range of precise modification strategies for MOFs such as surface microenvironment modulation, pore size and volume design, morphology tuning, and defect engineering. These strategies significantly improve enzymatic microenvironment, enzyme loading efficacy, and mass transfer. As‐designed MOF‐enzyme hybrids have exhibited outstanding performance across various domains, such as biosensing, biocatalysis, stimulus‐responsive delivery, and cancer therapy.

To further advance the applications of MOFs in enzyme immobilization, future efforts may be directed toward the following key areas: (1) It may be hard to co‐immobilize multi‐enzymes, integration of biomimetic catalytic MOFs for cascade catalysis will further expand the applicability of MOF‐enzyme hybrids. (2) Enzymes within cells are often localized in specific spatial arrangement, which significantly facilitates the efficiency of cascade reactions. MOFs with heterogeneous hierarchical porosity hold great potential as platforms for spatially controlled distribution of multiple enzymes with high precision. (3) In enzymatic catalysis, enzymes can interact with effector molecules at sites distinct from the active center to modulate their activity, a phenomenon known as allosteric regulation. Metastable MOFs allow flexible conformation of enzymes, and the permeability of effector molecules may enable the development of tunable activity‐regulation systems. (4) Current researches on MOF‐immobilized enzymes primarily focus on enhancing enzyme stability and catalytic efficiency, with limited consideration given to enzyme kinetics. In fact, reaction kinetics may take precedence in certain cases, such as detoxification. Designing MOF‐enzyme hybrids capable of binding substrate tightly at a low substrate concentration would be highly beneficial for detoxification applications. (5) The majority of enzymes immobilized in MOFs, such as GOx and HRP, have been extensively studied. Some enzymes, such as radical *S*‐adenosyl‐l‐methionine exhibit catalytic diversity but are inherently fragile. For enzymes with multi‐subunit or cofactors, such as oxidases, encapsulation or pore infiltration can provide enzymes with better protection from radicals during the reaction process. Exploring the immobilization of these valuable enzymes can broaden their applications. (6) Despite the development of numerous MOF‐enzyme hybrids, the fundamental molecular interactions between MOFs and enzymes remain poorly understood. Advanced atomic characterization, such as Raman spectroscopy and NMR, can offer valuable insights into these interactions and, in turn, optimize the immobilization process. (7) In the context of MOF‐based enzyme immobilization, machine learning offers a powerful approach to enhance the encapsulation efficiency, catalytic activity, and recyclability of immobilized enzymes,^[^ ^161^, ^162^, ^163^ ^]^ holding great promise for enabling comprehensive prediction and rational design of MOF‐immobilized enzyme systems. Overall, MOF‐based enzyme immobilization has demonstrated promising roles in many fields. Despite these advancements, concerns regarding MOF biocompatibility and toxicity necessitate comprehensive in vivo studies to evaluate long‐term safety profiles. Addressing these limitations demands innovative solutions, including the incorporation of biocompatible materials, stimulus‐responsive systems, and enhanced immobilization techniques. Integration with nanotechnology, targeted delivery systems, and emerging tools such as 3D printing and artificial intelligence can further optimize MOF‐enzyme hybrid design, facilitating their broader applications in environmental remediation, food security, bioanalysis, and energy conversion.

## Conflict of Interest

The authors declare no conflict of interest.

## Author Contributions

**Xiang Xu**: conceptualization (lead); writing—original draft (lead); and writing—review and editing (lead). **Jiacheng Wan**: conceptualization (equal); writing—original draft (supporting); and writing—review and editing (equal). **Jun Sun**: conceptualization (equal); validation (equal); and supervision (lead). **Lina Wu**: funding acquisition (supporting); resources (equal); supervision (equal); and validation (equal). **Jianping Lei**: funding acquisition (equal); writing—original draft (supporting); writing—review and editing (equal); and validation (equal).
